# Potent and Selective Inhibitors of Human Monoamine Oxidase A from an Endogenous Lichen Fungus *Diaporthe mahothocarpus*

**DOI:** 10.3390/jof7100876

**Published:** 2021-10-18

**Authors:** Geum Seok Jeong, Prima F. Hillman, Myung-Gyun Kang, Sungbo Hwang, Jong-Eun Park, Sang-Jip Nam, Daeui Park, Hoon Kim

**Affiliations:** 1 Department of Pharmacy, Research Institute of Life Pharmaceutical Sciences, Sunchon National University, Suncheon 57922, Korea; fever41@naver.com (G.S.J.); park140201@naver.com (J.-E.P.); 2 Department of Chemistry and Nanoscience, Ewha Womans University, Seoul 03760, Korea; primafitriah@gmail.com (P.F.H.); sjnam@ewha.ac.kr (S.-J.N.); 3 Department of Predictive Toxicology, Korea Institute of Toxicology, Daejeon 34114, Korea; myung-gyun.kang@kitox.re.kr (M.-G.K.); sungbo.hwang@kitox.re.kr (S.H.); daeui.park@kitox.re.kr (D.P.)

**Keywords:** endogenous lichen fungus, *Diaporthe mahothocarpus*, alternariol, 5-hydroxy-alternariol, mycoepoxydiene, selective monoamine oxidase A inhibitor, docking simulation

## Abstract

Using 126 endogenous lichen fungus (ELF) extracts, inhibitory activities against monoamine oxidases (MAOs) and cholinesterases (ChEs) were evaluated. Among them, extract ELF29 of the endogenous fungus *Diaporthe mahothocarpus* of the lichen *Cladonia symphycarpia* showed the highest inhibitory activity against hMAO-A. Compounds alternariol (AT), 5′-hydroxy-alternariol (HAT), and mycoepoxydiene (MED), isolated from the extract, had potent inhibitory activities against hMAO-A with IC_50_ values of 0.020, 0.31, and 8.68 µM, respectively. AT, HAT, and MED are reversible competitive inhibitors of hMAO-A with K_i_ values of 0.0075, 0.116, and 3.76 µM, respectively. The molecular docking studies suggested that AT, HAT, and MED had higher binding affinities for hMAO-A (−9.1, −6.9, and −5.6 kcal/mol, respectively) than for hMAO-B (−6.3, −5.2, and −3.7 kcal/mol, respectively). The relative tight binding might result from a hydrogen bond interaction of the three compounds with a Tyr444 residue in hMAO-A, whereas no hydrogen bond interaction was proposed in hMAO-B. In silico pharmacokinetics, the three compounds showed high gastrointestinal absorption without violating Lipinski’s five rules, but only MED showed high probability to cross the blood–brain barrier. These results suggest that AT, HAT, and MED are candidates for treating neuropsychiatric disorders, such as depression and cardiovascular disease.

## 1. Introduction

Lichens, called the clothes of the earth, are complex organisms in which green or blue-green algae have a symbiotic relationship with fungi. Lichens have been used as a folk remedy for centuries but have been less studied than single microorganisms [1]. Lichens are found in various environments, from temperate regions and tropical rainforests to extremely cold regions, such as deserts, tundra, and volcanoes; it is predicted that the materials they produce help them adapt to the environment [2]. Their primary and secondary metabolites exhibit diverse biological activities, such as antibiotic, antifungal, antiviral, anti-inflammatory, analgesic, antipyretic, antiproliferative, cytotoxic, and antioxidant effects [3,4]. Additionally, various studies have been conducted to develop new drugs rules, but only MED had blood–brain barrier permeability [5,6].

Depression is a phenomenon that causes sadness, despair, and discouragement due to a depressed mood or decreased interest and pleasure, and suicidal ideation when the symptoms occur severely [7]. Hundreds of millions of people have the disease, and the number is increasing yearly. It is known that the decrease of monoamines, which are neurotransmitters, such as norepinephrine, serotonin, and melatonin, within the nerve synapse, causes depression [8]. Alzheimer disease (AD), which can cause 60–70% of dementia, is a neurodegenerative disease that progresses slowly and gets worse. It was named after the German psychiatrist and pathologist Alois Alzheimer in 1906 [9]. As AD advances, it presents with a variety of symptoms, including language problems, disorientation, mood swings, and loss of motivation [10]. It is known that among the causes of AD is the excessive accumulation of a small peptide called β-amyloid (Aβ). Furthermore, excessive activity of monoamine oxidase (MAO) and cholinesterase (ChE) has been shown to be associated with AD.

MAO is an enzyme that oxidatively deaminates monoamines, which are neurotransmitters, and is classified into two isoforms, A and B [11]. Deamination of monoamine by MAO causes various diseases, depending on the isoforms type. While MAO-A is associated with neuropsychiatric disorders, such as depression and cardiovascular disease, MAO-B is associated with neurodegenerative diseases, such as AD and Parkinson disease (PD) [12]. Therefore, MAO inhibitors are used as therapeutic agents for neuropsychiatric and neurodegenerative diseases, respectively. ChEs, distinguished into acetylcholinesterase (AChE) and butyrylcholinesterase (BChE), are characterized by the degradation of choline; AChE degrades acetylcholine (ACh) into acetate and choline [13], and BChE degrades butyrylcholine (BCh) into butyrate and choline [14]. ACh is among the neurotransmitters, and ACh is most distributed in the cerebral cortex. AD patients have low ACh levels and high concentrations of ChE in the brain. This indicates that ChEs are one of the main causes of Alzheimer disease and that ChEs’ inhibitors are used as therapeutic agents for AD patients [15]. Additionally, BChE levels in the brains of AD patients appear to be significantly increased [16]. On the other hand, an enzyme called β-secretase (BACE-1) cleaves the β-site of amyloid precursor protein (APP) to produce and aggregate Aβ to induce accumulation in the brain, thereby affecting the progression of AD [17].

In a previous study, we reported that 5-hydroxy-2-methyl-chroman-4-one was isolated from an endogenous lichen fungus (ELF) extract, and it had selective inhibition of hMAO-B [6]. Here, the inhibitory activities of MAOs and ChEs against a library of 126 endogenous lichen extracts (ELFs) were evaluated. Among them, the ELF29 extract of the endogenous fungus *Diaporthe mahothocarpus* of the lichen *Cladonia symphycarpia* showed the highest hMAO-A inhibitory activity. Through activity-guided screening, alternariol (AT, **1**), 5’-hydroxy-alternariol (HAT, **2**), and mycoepoxydiene (MED, **3**) were isolated and identified from ELF29. Additionally, ChEs’ and BACE-1 inhibitory activities were measured for the compounds.

AT is a mycotoxin produced by *Alternaria* fungi and has been reported to have estrogenic and immunomodulatory effects [18,19] and can form reactive oxygen species (ROS) and interact with topoisomerase II relating to toxicity at >10 µM [20]. Additionally, it was known to have inhibitory activity on MAO-A and AChE [21]. However, the work was performed using rat brain extracts, unpurified enzymes. HAT is a known compound [22], but little information is available about HAT, except for applications in patents as antioxidants and cosmetics [23]. MED has various biological effects, including antimicrobial [24], osteoporosis relief [25], anticancer [26,27,28], and anti-inflammatory [29]. However, inhibitory activities of HAT and MED against hMAO-A have not been reported. In this study, the inhibitory abilities of AT, HAT, and MED against hMAO-A, hMAO-B, AChE, BChE, and BACE1 were investigated using purified enzymes; also, performed kinetics, reversibility experiments, and docking simulations were conducted for hMAO-A and hMAO-B, and in silico pharmacokinetic analyses were performed.

## 2. Materials and Methods

### 2.1. Evaluation of Enzyme Inhibitory Activities of ELF Extracts

A library of 126 extracts with ethyl acetate or butanol from ELF from Korea, China, and Antarctica was obtained from the Korea Lichen Research Institute (KOLRI) at Suncheon National University, Republic of Korea, for evaluation of the inhibitory activities of MAOs’ and ChEs’ enzymes. The extract was dissolved in DMSO at a concentration of 10 mg/mL and used for analysis. Final concentration of DMSO in the assay mixture was 0.2%, in which it did not inhibit the enzymes. All other chemicals and enzymes were purchased from Sigma-Aldrich (St. Louis, MO, USA).

### 2.2. MAO Activity Assay

Human recombinant MAO-A and MAO-B were assayed in a reaction mixture of 0.5 mL containing 50 mM sodium phosphate buffer (pH 7.2) and the substrates (0.06 mM kynuramine for hMAO-A and 0.3 mM benzylamine for hMAO-B). The hMAO-A and hMAO-B activities were continuously assayed by UV absorbance analysis at 316 nm and 250 nm, respectively, in kinetic mode for 30 min at room temperature. [30,31]. The reactions were started with the addition of substrates without preincubation of the enzyme and inhibitor.

### 2.3. ChE Activity Assay

AChE from electric eel and BChE from horse serum were used for cholinesterases and assayed with a slight modification of the Ellman method [32,33]. Shortly, after pre-incubating the enzyme and inhibitor for 15 min in 100 mM of sodium phosphate buffer (pH 7.5), 0.5 mM acetylthiocholine iodide (ATCI) or butyrylthiocholine iodide (BTCI) was added as a substrate for AChE or BChE assay, respectively. Then, 0.5 mM 5,5-dithiobis (2-nitrobenzoic acid) (DTNB) was added for color development for a final volume of 0.5 mL. Enzyme activity was measured for 15 min at room temperature in a kinetic mode at 412 nm.

### 2.4. BACE1 Activity Assay

BACE1 activity was analyzed using a BACE1 activity detection kit with fluorescence (Sigma-Aldrich, St Louis, MO, USA) and a spectrofluorometer (FS-2, Scinco, Seoul, Korea), measured under the conditions of excitation 320 nm and emission 405 nm at 37 °C for 2 h [6,34].

### 2.5. Culture and Extraction of ELF29

The fungus strain ELF29 was cultured in 80 2.8-L Fernbach flasks, each containing 1 L of potato dextrose broth (PDB) medium at 27 °C for 7 days with shaking at 120 rpm. The culture broth was extracted with the same volume, i.e., 80 L of ethyl acetate, and the solvent was evaporated *in vacuo* to yield 10.0 g of ELF29 crude extract.

### 2.6. Isolation of Compounds from ELF29 Crude Extract

The crude extract of ELF29 was fractionated by reversed-phase, open-column chromatography on C-18 resin with a step gradient of water and methanol to afford nine fractions. The fourth fraction was purified by HPLC (Waters 996 PDA Detector, Waters Corp, Milford, MA, USA) equipped with a reversed-phase column (Phenomenex Luna C-18 (2), 250 × 100 mm, 5 μm, 100 Å, 2.0 mL/min, UV = 254 nm; Torrance, CA, USA) using an isocratic solvent system from 40% CH_3_CN in water to yield alternariol (AT, **1**, 4.2 mg, *t*_R_ = 26.2 min, purity = 99.3%), 5′-hydroxy-alternariol (HAT, **2**, 2.0 mg, *t*_R_ = 18.6 min, purity = 99.3%), and mycoepoxydiene (MED, **3**, 30.2 mg, *t*_R_ = 22.9 min, purity = 99.2%). The purities of the compounds were calculated by HPLC chromatograms (Supplementary Figures S1–S3).

### 2.7. Structure Analysis of the Compounds through NMR and LC/MS

Low-resolution LC/MS measurements were performed using the Agilent Technologies’ 1260 quadrupole (Agilent Technologies, Santa Clara, CA, USA) and Waters Micromass-ZQ 2000 MS system (Waters Corp) using a reversed-phase column (Phenomenex Luna C18 (2), 50 mm × 4.6 mm, 5 µm, 100 Å) at a flow rate of 1.0 mL/min at the National Research Facilities and Equipment Center (NanoBioEnergy Materials Center) at Ewha Womans University. The ^1^H and 2D NMR spectra were recorded at 500 MHz in DMSO-*d_6_* and CD_3_OD_,_ using solvent signal as internal standard on Varian Inova spectrometers (Bruker, Billerica, MA, USA). The ^13^C NMR spectra were acquired at 125 MHz on the Varian Inova spectrometer. The data were provided in Supplementary (Figures S4–S18).

### 2.8. Analysis of Inhibitory Activities and Kinetics of the Compounds

Assays for inhibitory activities of compounds against hMAO-A, hMAO-B, AChE, BChE, and BACE1 were performed at 10-µM concentration of inhibitor. IC_50_ values of the compounds were determined using several concentrations of inhibitors, and K_i_ values were determined using a Lineweaver–Burk plot and its secondary plots of the slope vs. inhibitor concentration at three concentrations of inhibitors, i.e., ~1/2 × IC_50_, ~IC_50_, and ~2 × IC_50_ [35].

### 2.9. Analysis of Inhibitor Reversibility

The reversibility analyses of the compounds were performed at ~2 × IC_50_ concentration of the inhibitor, and a recovery experiment was subjected through dialysis in 50 mM sodium phosphate buffer (pH 7.2). Toloxatone as a reversible inhibitor and clorgyline as an irreversible inhibitor were used as references for hMAO-A [36].

### 2.10. Docking Simulations of the Compounds with hMAO-A and hMAO-B

AutoDock Vina [37], which has an automated docking facility, was used for docking simulations of the compounds to hMAO-A (PDB ID: 2Z5X) and hMAO-B (PDB ID: 3PO7). The PDB files were retrieved from Protein Data Bank (PDB) (www.rcsb.org, accessed on 16 July 2021) and prepared for docking simulation by removing the heteroatoms and water molecules. To locate the ligand-binding pocket for each enzyme, we used a set of the predefined active sites obtained from a complex of hMAO-A with 7-methoxy-1-methyl-9H-beta-carboline (HRM) and a complex of hMAO-B 1-(1,2-benzoxazol-3-yl)methanesulfonamide (ZON). For the docking simulation, we performed the following steps: creation of 2D structures of the compounds [37], conversion of the 2D structures into 3D structures [38], and energy minimization using the ChemOffice program (http://www.cambridgesoft.com, accessed on 16 July 2021) [39]. Docking simulations of hMAO-A and hMAO-B with the compounds were performed using AutoDock Vina [38]. From the docking results, we checked for possible hydrogen bonding using relaxation constraints of 0.4 Å and 20.0° using FindHBond program in UCSF Chimera [39]. To determine the predicted binding poses of AT for hMAO-A or hMAO-B, we performed molecular dynamics based on nanoscale molecular dynamics (NAMD) [40] and visual molecular dynamics (VMD) [41] software. The compound was simulated under the condition of the complex with hMAO-A or hMAO-B, using CHARMM parameters [42]. The water solvent model was used for the solvation box plug-in provided by VMD. To consider stable water molecules located in the close surface of protein, the size of water box was the protein size plus 5 Å. The time step and number of minimizations were 1.0 fs and 100,000 step, respectively, and the total running time was 1 ns. The molecular dynamics were examined on the basis of Root Mean Square Deviation (RMSD) during the time. The structure variation was calculated by RMSD values of protein–ligand complexes from 0 to 1 ns.

### 2.11. Analysis of Pharmacokinetic and Physicochemical Parameters of the Compounds Using in Silico Method

Analysis of the pharmacokinetic and physicochemical parameters of the compounds was performed using the SwissADME web tool (http://www.swissadme.ch/, accessed on 30 July 2021). Gastrointestinal absorption, blood–brain barrier (BBB) permeability, p-glycoprotein substrate, cytochrome p inhibitory ability, physicochemical parameters, and Lipinski violation were analyzed [43].

## 3. Results

### 3.1. Inhibitory Activities of ELF Extracts against hMAO-A, hMAO-B, AChE, and BChE

Inhibitory activities against hMAO-A, hMAO-B, AChE, and BChE were evaluated with 126 extracts of ELF from Korea, China, and Antarctica at a concentration of 20 µg/mL. Based on the cutoff values of residual activity, i.e., 20% and 30% for MAOs and ChEs, respectively, eight extracts for hMAO-A, nine for hMAO-B, and four for BChE were selected (Table 1). ELF21, ELF24, ELF29, ELF93, and ELF114 were potent against hMAO-A and hMAO-B. In this study, ELF21, ELF24, ELF29, and ELF93 were selected as the highest candidates for isolation of potent hMAO-A inhibitors. However, ELF24 was limited in its storage at KOLRI. Three strains of ELF21, ELF29, and ELF93 were cultured to confirm the reproducibility, and it was found that ELF29 showed reproducibly potent inhibitory activity against hMAO-A (Table 2). That is, ELF29 showed 7.25% and 10.7% residual activities against hMAO-A in the original and cultured extracts, whereas the cultured extract showed much higher residual activity against hMAO-B (53.4%) than the original extract (10.8%) (Table 2). The other two strains, ELF21 and ELF93, exhibited much lower inhibitory activities than those of their original extracts. Therefore, ELF29 was selected and used for further study. ELF29 was identified as an endogenous fungus *Diaporthe mahothocarpus* of the lichen *Cladonia symphycarpia*. 

### 3.2. Isolation and Identification of the Compounds from the ELF29 Extract

**Figure 1:**
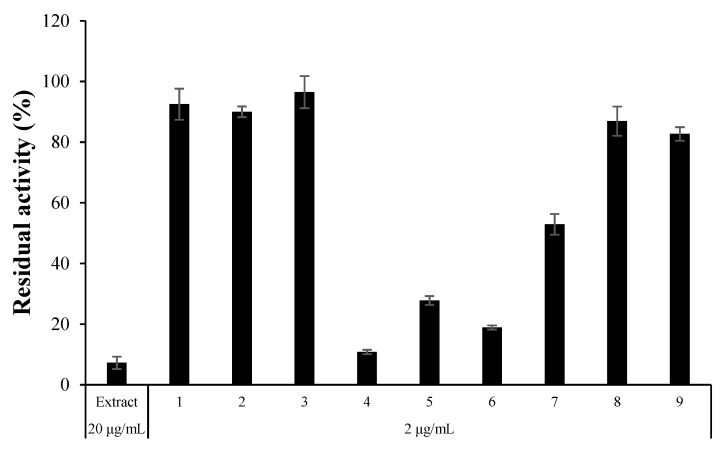
Residual activity of the ELF29 extract and nine fractions collected from the C18 column chromatography. Residual activities of the extract and the fractions were measured at 20 and 2 µg/mL, respectively.

**Figure 2:**
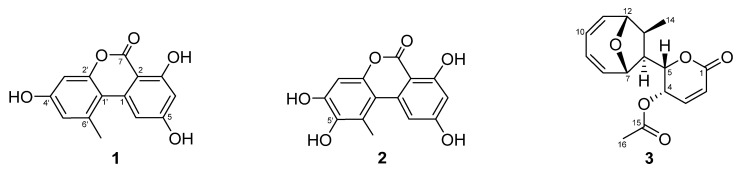
Chemical structures of alternariol (AT) (**1**), 5′-hydroxy-alternariol (HAT) (**2**), mycoepoxydiene (MED) (**3**).

Culture broth of ELF29 was extracted with EA, and its constituents were separated into nine fractions through a C18 column, and the fourth fraction showed the highest inhibitory activity against hMAO-A (Figure 1). The fraction was further separated into 12 single compounds. Among them, three compounds, 4, 7, and 8, were potent hMAO-A inhibitors, and their structures were identified as AT (**1**), HAT (**2**)**,** and MED (**3**) (Table 3, Figure 2). However, structures of the rest of the compounds could not be determined due to the limited amount, and also some compounds were slowly decomposed.

Compound **1** was isolated as a dark-yellow oil and revealed the *m/z* = 259.05 [M+H]^+^ in LRESIMS spectroscopic data. The ^1^H NMR spectrum of **1** displayed two sets of meta-coupled aromatic protons [*δ*_H_ 7.26 (d, *J* = 2.0 Hz, H-6), 6.37 (d, *J* = 2.0 Hz, H-4), 6.70 (d, *J* = 2.0 Hz, H-5′), 6.61 (d, *J* = 2.0 Hz, H-3′)], and one methyl singlet [*δ*_H_ 2.76 (s, 6′-CH_3_)]. The ^13^C NMR spectrum of **1** had 14 carbons and HSQC spectra illustrated one carbonyl carbon [*δ*_C_ 166.8 (C-7)], eight fully substituted carbons [*δ*_C_ 166.9 (C-5), 166.2 (C-3), 159.8 (C-4′), 154.4 (C-2′), 140.0 (C-1), 139.8 (C-6′), 110.9 (C-1′), 99.1 (C-2)], four methine carbons [*δ*_C_ 118.5 (C-5′), 105.4 (C-6), 102.7 (C-3′), 101.9 (C-4)], and one methyl singlet carbon [*δ*_C_ 25.8 (6′-CH_3_)]. Compound **1** was identified as alternariol based on the comparison of NMR data in the literature [44]. 

Compound **2** was isolated as a dark-yellow oil and revealed the *m/z* = 275.23 [M+H]^+^ in LRESIMS spectroscopic data. The ^1^H NMR spectrum of **2** was almost identical to that of 1, except for the absence of one aromatic proton. The 16 amu difference in MS data and the de-shield shift of C-5′ indicated the substitution of a hydroxy group at C-5 in the structure. Thus, compound **2** was determined as 5′-hydroxy-alternariol [22].

Compound **3** was isolated as white, feather-like crystals and revealed the *m/z* = 291.32 [M+H]^+^ in LRESIMS spectroscopic data. The ^1^H NMR spectrum of **3** exhibited six olefinic protons [*δ*_H_ 7.01 (dd, *J* = 6.1, 9.6 Hz, H-3), 6.18 (d, *J* = 9.6 Hz, H-2), 6.05 (m, H-11), 5.91 (m, H-8), 5.81 (m, H-10), 5.78 (m, H-9)], one methyl singlet [*δ*_H_ 1.93 (s, H-16)], and one methyl doublet [*δ*_H_ 0.97 (d, *J* = 6.7 Hz, H-14)]. The ^13^C NMR and HSQC spectra displayed two carbonyls [*δ*_C_ 170.1 and 162.7], six olefinic methine groups [*δ*_C_ 141.4, 138.3, 138.3, 126.0, 124.8, and 124.7], four oxygenated methine groups [*δ*_C_ 86.0, 77.4, 75.3, and 63.0], two methine groups [*δ*_C_ 52.8 and 50.2], and two methyl groups [*δ*_C_ 20.9 and 14.3]. By comparing the NMR data of **3** to those of previously reported ones, **3** was identified as mycoepoxydiene [45].

AT (**1**): ^1^H (500 MHz, CD_3_OD); δ_H_ 11.90 (s, 3-OH), 7.26 (d, *J* = 2.0 Hz, H-6), 6.70 (d, *J* = 2.0 Hz, H-5′), 6.61 (d, *J* = 2.0 Hz, H-3′), 6.37 (d, *J* = 2.0 Hz, H-4), 2.76 (s, 6′-CH_3_), ^13^C NMR (125 MHz, CDOD_3_); δ_C_ 166.9 (C-5), 166.8 (C-7), 166.2 (C-3), 159.8 (C-4′), 154.4 (C-2′), 140.0 (C-1), 139.8 (C-6′), 118.5 (C-5′), 110.9 (C-1′), 105.4 (C-6), 102.7 (C-3′), 101.9 (C-4), 99.1 (C-2), 25.8 (6′-CH_3_), LR-ESI-MS *m/z* = 259.23 [M+H]^+^

HAT (**2**): ^1^H (500 MHz, DMSO-d_6_); δ_H_ 11.89 (s, 3-OH), 8.64 (s, 5′-OH), 7.29 (d, *J* = 2.0 Hz, H-6), 6.71 (s, H-3′), 6.35 (d, *J* = 2.0 Hz, H-4), 2.59 (s, 6′-CH_3_), ^13^C NMR (125 MHz, DMSO-d_6_); δ_C_ 164.9 (C-5), 165.2 (C-7), 164.0 (C-3), 147.5 (C-4′), 144.8 (C-2′), 141.5 (C-5′), 138.6 (C-1), 121.8 (C-6′), 109.0 (C-1′), 104.3 (C-6), 100.7 (C-3′), 100.7 (C-4), 97.7 (C-2), 25.8 (6′-CH_3_), LR-ESI-MS *m/z* = 275.23 [M+H]^+^

MED (**3**): ^1^H (500 MHz, DMSO-d_6_); δ_H_ 7.01 (dd, *J* = 6.1, 9.6 Hz, H-3), 6.18 (d, *J* = 9.6 Hz, H-2), 6.05 (m, H-11), 5.91 (m, H-8), 5.81 (m, H-10), 5.78 (m, H-9), 5.18 (dd, J = 2.2, 6.1 Hz, H-4), 4.60 (dd, *J* = 2.2, 11.4 Hz, H-5), 4.39 (t, *J* = 6.4 Hz, H-7), 4.19 (d, *J* = 4.5 Hz, H-12), 2.75 (m, H-13), 2.73 (m, H-6), 1.93 (s, H-16), 0.97 (d, *J* = 6.7 Hz, H-14), ^13^C NMR (125 MHz, DMSO-d_6_); δ_C_ 170.1 (C-15), 162.7 (C-1), 141.4 (C-3), 138.3 (C-11), 138.3 (C-8), 126.0 (C-9), 124.8 (C-2), 124.7 (C-10), 86.0 (C-12), 77.4 (C-5), 75.3 (C-7), 63.0 (C-4), 52.8 (C-13), 50.2 (C-6), 20.9 (C-16), 14.3 (C-14), LR-ESI-MS *m/z* = 291.32 [M+H]^+^

### 3.3. Inhibitory Activities of the Isolated Compounds against MAOs, ChEs, and BACE1

The inhibitory activity of the three compounds against hMAO-A, hMAO-B, AChE, and BChE was analyzed at a concentration of 10 µM. Additionally, BACE1 inhibitory activity was evaluated. All three compounds had effective inhibitory activity against hMAO-A with <50% residual activity, and AT and HAT strongly inhibited hMAO-A (Table 4). AT strongly inhibited hMAO-A (IC_50_ = 0.020 µM) and moderately inhibited hMAO-B (IC_50_ = 20.7 µM) with a selectivity index (SI) of 1035. HAT had an IC_50_ value of 0.31 µM for hMAO-A and an SI of >129.0 for hMAO-A over hMAO-B. AT and HAT exhibited higher potency for hMAO-A than a reference compound, toloxatone (IC_50_ = 1.1 µM). Additionally, MED effectively inhibited hMAO-A (IC_50_ = 8.7 µM). In addition, all three compounds showed some inhibitory activity on AChE (AT: IC_50_ = 10.0 μM; HAT: IC_50_ = 19.9 μM; MED: IC_50_ = 18.6 μM) but were ineffective in BChE inhibition (Table 4). Interestingly, HAT showed slight inhibitory activity against BACE1 (IC_50_ = 24.7 µM) (Table 4). Conclusively, the isolated compounds AT, HAT, and MED were confirmed to be effective and selective inhibitors of hMAO-A.

### 3.4. Kinetic Studies

**Figure 3:**
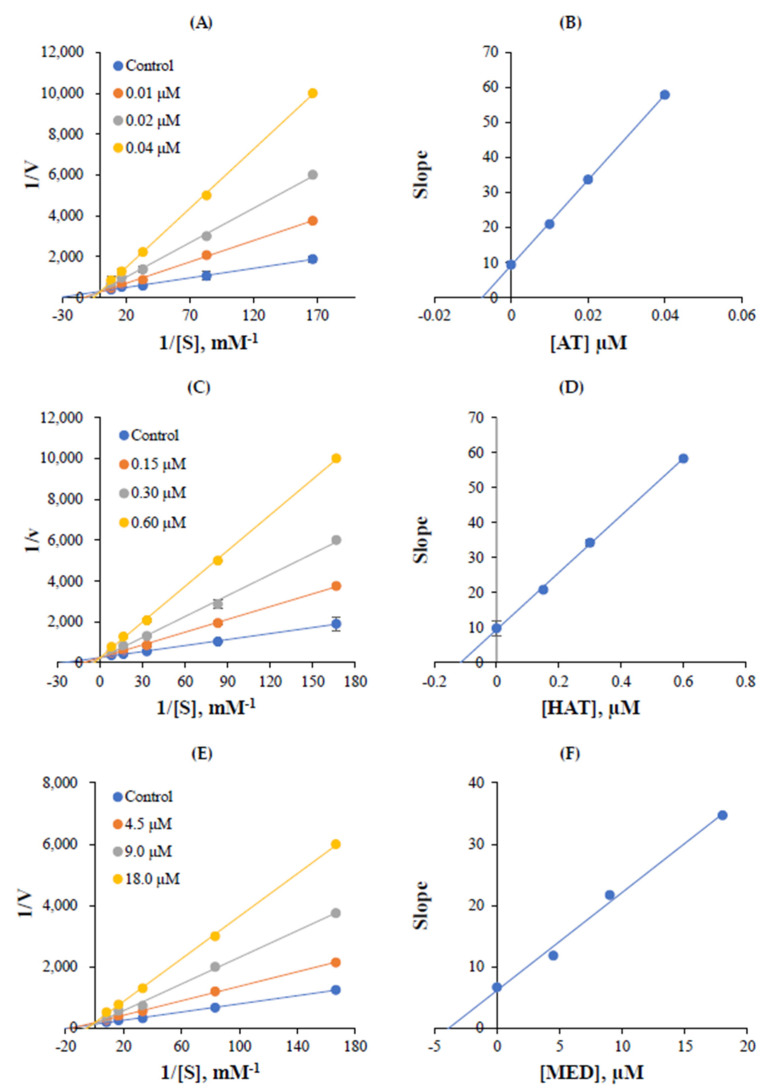
Lineweaver–Burk plots (**A**,**C**,**E**) for hMAO-A inhibition by AT, HAT, and MED, and their secondary plots (**B**,**D**,**F**) using their slopes following inhibitor concentrations. Five different substrate concentrations (0.006, 0.012, 0.03, 0.06, and 0.12 µM) were used, and inhibitors were added to make three concentrations, i.e., ~1/2 × IC_50_, ~IC_50_, and ~2 × IC_50_. Triplicate experiments determined error bars.

The inhibition mode of AT, HAT, and MED for hMAO-A was analyzed using the Lineweaver–Burk plot. The hMAO-A inhibition plots of AT crossed at the y-axis (Figure 3A), and the secondary plot obtained using the inhibitor concentrations vs. their slopes showed a K_i_ value of AT to be 0.0075 ± 0.0007 µM for hMAO-A inhibition (Figure 3B). Similar to AT, the inhibition plots of HAT crossed at the y-axis (Figure 3C), and a K_i_ value from the secondary plot was 0.116 ± 0.016 µM (Figure 3D). MED also crossed at the y-axis, and a Ki value was 3.76 ± 0.07 µM (Figure 3E,F). These results indicated that the three compounds are competitive inhibitors of hMAO-A.

### 3.5. Reversibility Analysis of hMAO-A Inhibition by the Compounds

**Figure 4:**
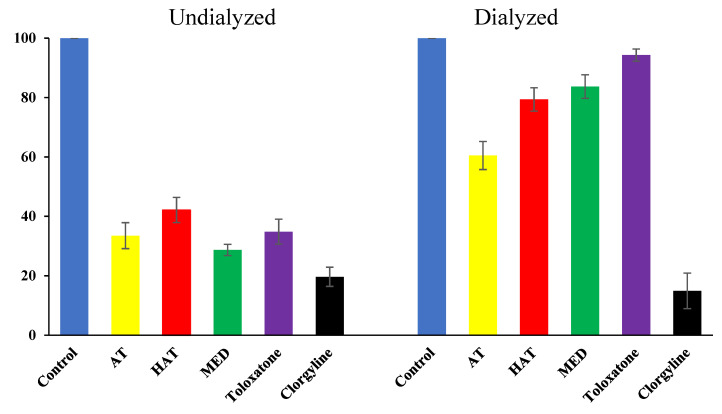
Recovery of hMAO-A inhibition by AT, HAT, and MED through dialysis. The concentrations of inhibitors used in the experiment were ~ 2 × IC_50_ values. AT, 0.040 µM; HAT, 0.60 µM; MED, 18.0 µM; toloxatone 2.0 µM; clorgyline 0.014 µM. Toloxatone and clorgyline were used as references for reversible and irreversible inhibitors of hMAO-A, respectively.

The reversibility test of hMAO-A inhibition by AT, HAT, and MED was conducted through a recovery test after dialysis. Inhibition of hMAO-A by AT was restored from 33.5% to 60.5% through dialysis, based on the residual activity (Figure 4). Inhibition by HAT was also recovered from 42.1% to 79.4%, and that of MED recovered from 28.7% to 83.7%. Toloxatone, known as a reversible inhibitor of hMAO-A, also showed almost complete recovery (34.8% to 94.3%) (Figure 4). Contrarily, the irreversible inhibitor clorgyline showed no recovery at all (19.7% to 14.9%). These results show that AT, HAT, and MED are reversible inhibitors of hMAO-A.

### 3.6. Molecular Docking Simulation

**Figure 5:**
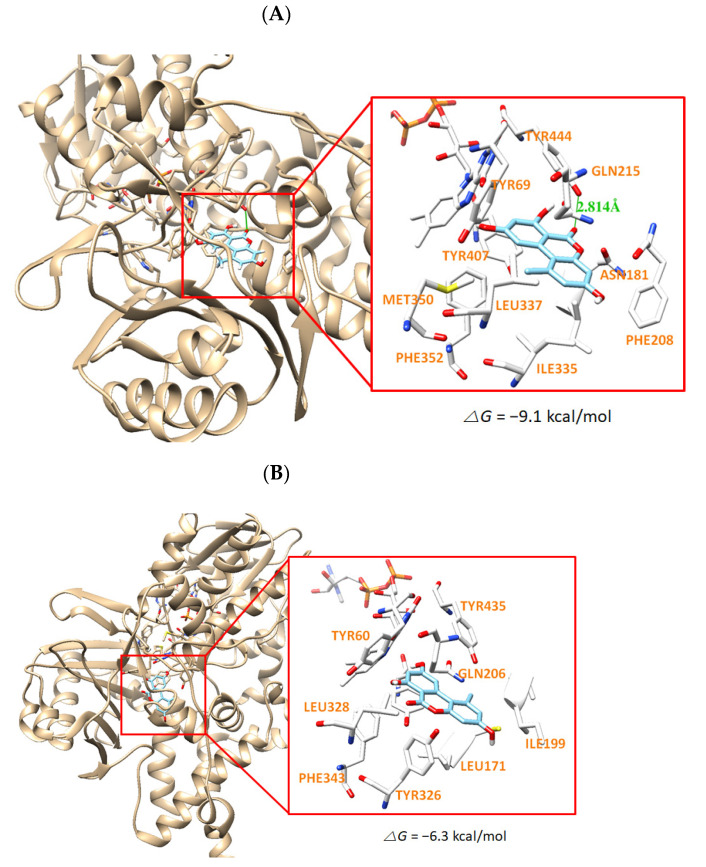
The docking simulation of AT with hMAO-A (2Z5X) (**A**) and hMAO-B (3PO7) (**B**) as determined by the UCSF Chimera. AT interacts through a hydrogen bond with Tyr444 residue of hMAO-A at a distance of 2.814 Å.

**Figure 6:**
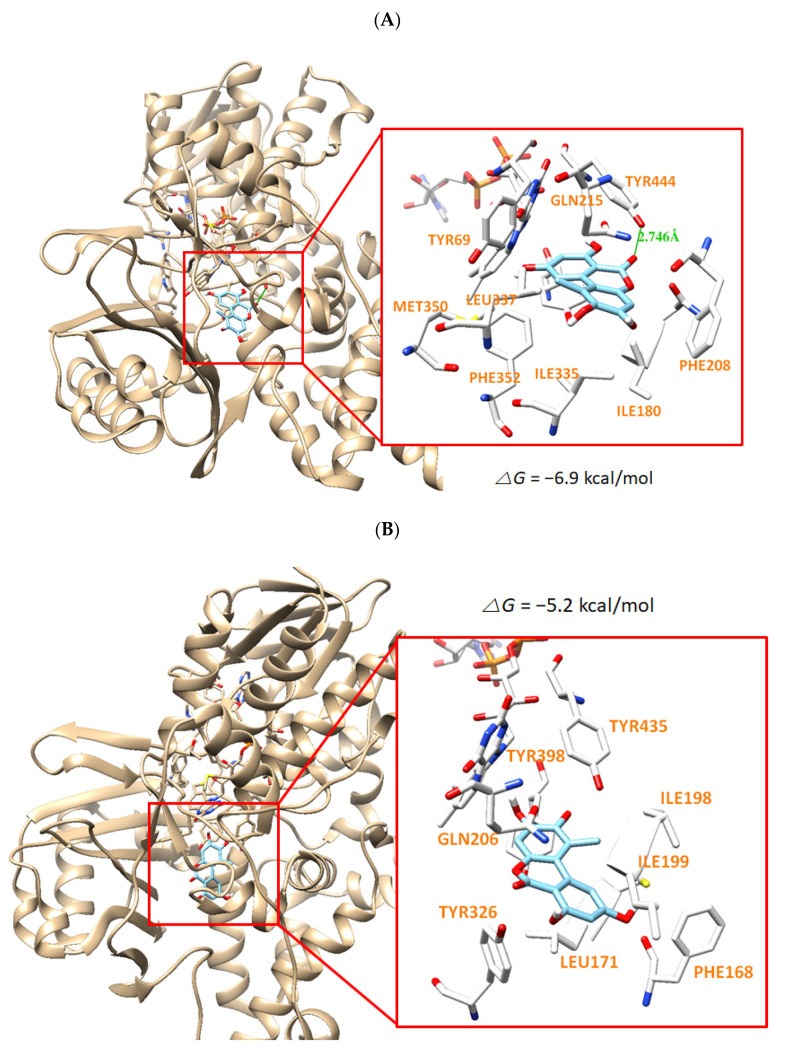
The docking simulation of HAT with hMAO-A (2Z5X) (**A**) and hMAO-B (3PO7) (**B**) as determined by the UCSF Chimera. HAT interacts through a hydrogen bond with Tyr444 residue of hMAO-A at a distance of 2.764 Å.

**Figure 7:**
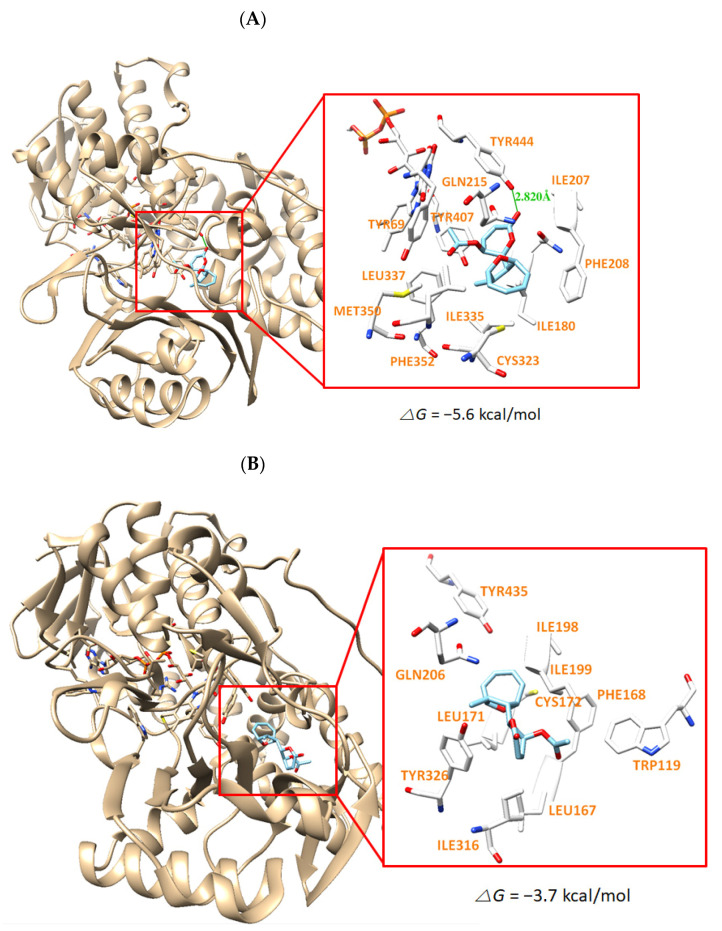
The docking simulation of MED with hMAO-A (2Z5X) (**A**) and hMAO-B (3PO7) (**B**) as determined by the UCSF Chimera. MED interacts through a hydrogen bond with Tyr444 residue of hMAO-A at a distance of 2.820 Å.

The docking simulation results showed that the compounds were properly located within the binding site of HRM with hMAO-A (PDB: 2Z5X) or of ZON with hMAO-B (PDB ID: 3PO7). The docking poses and the binding scores of the compounds AT, HAT, and MED with hMAO-A or hMAO-B were presented in Figure 4, Figure 5 and Figure 6, respectively. From the simulation, it was confirmed that the binding energies of AT, HAT, and MED to hMAO-A were −9.1, −6.9, and −5.6 kcal/mol, respectively, and exceeded those of hMAO-B (−6.3, −5.2, and −3.7 kcal/mol, respectively). An intermolecular hydrogen bond interaction was predicted on Tyr444 residue in hMAO-A at distances of 2.814, 2.764, and 2.820 Å for AT, HAT, and MED, respectively, but not in hMAO-B (Figure 5, Figure 6 and Figure 7). The binding energies of all compounds sufficiently explain that AT was the most potent inhibitor of hMAO-A and three compounds inhibited selectively hMAO-A, which corresponded to the IC_50_ values of the inhibitory assay provided in Table 4.

In addition, the molecular dynamics for hMAO-A and hMAO-B complexes with AT were performed to confirm whether the calculated binding poses were stable. The RMSD values steadily increased from 0 to 200 ps and reached stable state throughout the simulation (Figure S19). The average RMSD values of AT for hMAO-A and hMAO-B were estimated to be 1.422 and 1.437 Å, respectively. The average RMSD of the hMAO-A and AT complex was shorter than that of hMAO-B and AT complex. During 0~100 ps, the RMSD values for hMAO-A and hMAO-B complexes with AT were 1.304 and 1.037 Å, respectively. The RMSD values steadily increased from 100 to 200 ps to a local energy minimum. During 200~1000 ps, the average RMSD values for hMAO-A and hMAO-B complexes with AT were 1.700 and 1.735 Å, respectively. As results, the differences in the RMSD values from the initials were 0.396 and 0.698 Å, respectively. It means that the complex of hMAO-A with AT was less movable than the complex of hMAO-B with AT. In addition, AT was located in the binding site of hMAO-A or hMAO-B during all periods. Furthermore, the complex for hMAO-A with AT showed two hydrogen-bonding interactions in the structure of the last frame of MD simulation, and the distance between AT and Tyr444 residue in hMAO-A was 1.984 Å, though a hydrogen-bond interaction (2.814 Å) was predicted in hMAO-A, not in hMAO-B, in the molecular docking simulation. That is, the distance between AT and Tyr444 of hMAO-A got shorter from 2.814 to 1.984 Å. Therefore, AT was predicted to have stronger bindings to hMAO-A than to hMAO-B, indicating that the molecular dynamics well supported the stable binding of AT to hMAO-A in this study.

### 3.7. In Silico Pharmacokinetic Analysis of the Compounds 

Due to the analysis of the compounds’ pharmacokinetics, using the SwissADME web tool, all three compounds showed high gastrointestinal absorption, and MED was permeable to BBB, but AT and HAT were not permeable (Table 5). AT was shown to inhibit CYP1A2 and CYP2D6, and HAT was shown to inhibit cytochrome P450 CYP1A2. It was also shown that MED inhibited no cytochrome P450 (Table 5). The results of the Lipinski parameters of the compounds were predicted not to violate Lipinski’s rule of five in all three compounds, AT, HAT, and MED (Table 6). These results may provide benefits when the compounds are used as central nervous system (CNS) drugs.

## 4. Discussion

In this study, inhibitory activities of 126 ELF extracts against MAOs and ChEs were evaluated. Among them, eight extracts for hMAO-A, nine extracts for hMAO-B, and four extracts for BChE were selected as effective candidates, but no extracts for AChE was selected. Based on reproducibility for large culture and novelty of strain, the extract of ELF29 was selected as a final source, and it was identified as an endogenous fungus *Diaporthe mahothocarpus*, living in a symbiotic relationship with the lichen *Cladonia symphycarpia*.

The diverse species of the genus *Cladonia* have various biological activities. Biruloquinone from *Cladonia macilenta* inhibits AChE [46], and fumarprotocetraric acid from *Cladonia verticillaris* has been confirmed to have expectorant and antioxidant properties [47]. Additionally, *Cladonia rangiformis* and *Cladonia convolute* extracts showed antiproliferative and apoptosis against breast cancer cells [48]. However, no inhibitory ability analysis for compounds from *Cladonia* against MAOs is currently available.

*Diaporthe* is a genus of endogenous filamentous fungal plant pathogens, in which some species of *Diaporthe* produce secondary metabolites that cause animal toxicity, such as sheep lupinosis [49]. Various species of *Diaporthe* have been reported, but *Diaporthe mahothocarpus* was identified recently [50], and little has been reported about its secondary metabolites and their biological activities, including MAO inhibitory activities.

Only a few MAO inhibitors have been isolated from lichens; an anthraquinone solorinic acid from a lichen *Solorina crocea* had MAO inhibitory activity (IC_50_ = 14.3 µM) [51], and hydroxy-2-methyl-chroman-4-one isolated from an ELF *Daldinia fissa* had MAO inhibitory activities (IC_50_ = 13.97 and 3.23 μM for hMAO-A and hMAO-B, respectively) [6]. Furthermore, 4-acylresorcinol, a synthetic derivative of the lichen compound, showed MAO inhibitory activity (IC_50_ = 4.27 μM) [52].

AT is a toxic metabolite of the *Alternaria* fungus [53]. Inhibitory activity on MAO-A and AChE using rat brain extracts was reported, and it was found that K_i_ values of AT for AChE and MAO were similar, i.e., from 1.6 to 2.1 mM and from 2.4 to 3.8 mM, respectively [21]. In this study, we used purified hMAO-A and AChE and found that AT was a potent and selective hMAO-A inhibitor (IC_50_ = 0.020 µM; K_i_ = 0.0075 µM) and showed moderate AChE inhibition (IC_50_ = 10.0 µM). These results differed significantly from those of the previous study, probably due to the presence of diverse components in the extracts, difference of enzyme sources, and difference of assay methods. Additionally, in this study, HAT was isolated, and it was observed that HAT had potent inhibitory activity against hMAO-A (IC_50_ = 0.31 µM; K_i_ = 0.116 µM). HAT is a rare compound, for which very little information is available. HAT was 5.5 times effective against hMAO-A than alternariol monomethyl ether (IC_50_ = 1.71 µM) [54]. Additionally, HAT effectively inhibited BACE1 (IC_50_ = 24.71 µM), similar to that of quercetin (IC_50_ = 20.46 µM), which is known as a BACE1 inhibitor. MED had effective hMAO-A inhibitory activity (IC_50_ = 8.68 μM), although it showed a lower inhibitory activity compared to AT and HAT.

Regarding the potency for hMAO-A, AT and HAT had 54.0 and 3.5 times, respectively, higher inhibitory activity than toloxatone (IC_50_ = 1.08 µM), a well-known reversible inhibitor of hMAO-A, although MED had 1.6 times lower inhibitory activity than toloxatone. Therefore, it is suggested that the three compounds have effective inhibitory activity against hMAO-A as natural compounds and have the potential to be used as lead compounds for the development of more efficient derivatives. Additionally, following the docking simulation, AT, HAT, and MED were predicted to form hydrogen bonds with Tyr444 of hMAO-A, whereas no hydrogen bonding was predicted with hMAO-B. The hydrogen bond is thought to contribute to the tighter interaction of the compounds with hMAO-A. Thus, the docking results produced a good correlation with the experimental data of the enzyme inhibition assays. Moreover, they do not violate Lipinski’s five rules and they have high gastrointestinal absorption, indicating the pharmacological potentials of the compounds. 

## 5. Conclusions

Among 129 ELF extracts, ELF29 showed the highest inhibitory activity against hMAO-A. After a large culture, AT, HAT, and MED were isolated and identified from ELF29 extract, and they showed strong inhibitory activity against hMAO-A with high SI values. HAT also exhibited effective inhibitory activity against BACE1 (IC_50_ = 24.7 µM). Furthermore, in silico pharmacokinetic analysis showed that they had high gastrointestinal absorption and kept Lipinski’s five rules, and MED had BBB permeability. These results suggest that AT, HAT, and MED are potent or moderate selective hMAO-A inhibitors and may be considered as candidates of new pharmaceutical development with improvement of BBB permeability.

## Figures and Tables

**Table 1:** Inhibitory activities of ELF extracts against hMAO-A, hMAO-B, AChE, and BChE.

ELF No.	Residual Activity at 20 µg/mL (%)			
	hMAO-A	hMAO-B	AChE	BChE
3	14.9 ± 1.91	25.2 ± 5.49	87.9 ± 4.29	95.2 ± 1.42
5	48.3 ± 0.48	68.9 ± 3.14	72.0 ± 9.02	14.5 ± 1.81
12	68.9 ± 0.96	13.5 ± 0.52	90.2 ± 1.24	72.8 ± 3.30
19	81.7 ± 4.84	10.9 ± 2.06	91.9 ± 0.84	93.5 ± 2.45
21	4.18 ± 2.69	−4.67 ± 1.32	63.3 ± 1.32	64.8 ± 1.20
23	80.1 ± 6.66	68.2 ± 6.61	86.6 ± 2.80	20.9 ± 1.84
24	7.25 ± 1.02	2.80 ± 0.42	56.6 ± 0.98	93.2 ± 6.70
28	35.1 ± 5.64	18.6 ± 0.001	95.7 ± 4.22	71.3 ± 0.24
29	7.25 ± 2.05	10.8 ± 1.39	94.3 ± 1.42	86.3 ± 2.92
45	14.8 ± 0.47	41.0 ± 2.26	91.1 ± 3.14	83.3 ± 0.00
49	15.0 ± 0.40	34.6 ± 5.27	97.1 ± 6.09	26.2 ± 1.59
50	50.0 ± 2.80	10.1 ± 2.26	94.4 ± 1.17	45.2 ± 2.96
54	71.4 ± 3.59	43.2 ± 5.41	86.8 ± 2.33	22.2 ± 0.50
93	9.83 ± 0.48	−1.70 ± 7.23	78.0 ± 4.09	56.1 ± 2.20
114	16.7 ± 0.55	15.5 ± 1.68	75.5 ± 6.43	97.2 ± 0.72

Results are expressed as mean and standard deviation from duplicate experiments.

**Table 2:** Inhibitory activities of the original and cultured extracts of ELF candidates against hMAO-A and hMAO-B.

ELF No.	Residual Activity at 20 µg/mL (%)			
	Original Extract		Cultured Extract	
	hMAO-A	hMAO-B	hMAO-A	hMAO-B
21	4.18 ± 2.69	−4.67 ± 1.32	>50	45.6 ± 8.71
29	7.25 ± 2.05	10.8 ± 1.39	10.7 ± 4.38	53.4 ± 10.1
93	9.83 ± 0.48	−1.70 ± 7.23	>50	>50

Results are expressed as mean and standard deviation from duplicate experiments.

**Table 3:** The hMAO-A inhibitory activity of isolated compounds from ELF29.

No.	Sample Name	Residual Activity at 2 µg/mL (%)	Name	MW
		hMAO-A		
1	LFF29-C18-40L-F4-1&2-T1	88.2 ± 3.07		
2	LFF29-C18-40L-F4-1&2-T2	85.5 ± 4.17		
3	LFF29-C18-40L-F4-1&2-T3	86.9 ± 0.47		
4	LFF29-C18-40L-F4-3	3.39 ± 3.43	5′-hydroxy-alternariol	274.23
5	LFF29-C18-40L-F4-3-1	44.9 ± 5.90		
6	LFF29-C18-40L-F4-3-2	82.0 ± 0.92		
7	LFF29-C18-40L-F4-4-4	66.7 ± 1.84	Mycoepoxydiene	290.32
8	LFF29-C18-40L-F4-4-5	−5.41 ± 0.98	Alternariol	258.05
9	LFF29-C18-40L-F4-4-6	14.7 ± 1.85		
10	LFF29-C18-40L-F4-4-7	46.6 ± 2.12		
11	LFF29-C18-40L-F4-4-8	90.5 ± 1.30		
12	LFF29-C18-40L-F4-4-9	80.8 ± 0.46		

Results are expressed as mean and standard deviation from duplicate experiments.

**Table 4:** Analysis of enzyme inhibitory activities of the isolated compounds.

	Residual Activity at 10 µM (%)				
	hMAO-A	hMAO-B	AChE	BChE	BACE1
AT ^a^	−3.1 ± 0.0	75.6 ± 2.3	58.7 ± 2.0	93.3 ± 4.1	80.9 ± 0.2
HAT ^a^	17.7 ± 1.1	83.3 ± 4.0	65.6 ± 3.8	96.2 ± 2.3	71.3 ± 3.8
MED	42.7 ± 0.9	85.4 ± 6.9	65.2 ± 2.7	95.5 ± 5.5	80.5 ± 2.7
	**IC_50_ (µM)**				**SI ^b^**
	**hMAO-A**	**hMAO-B**	**AChE**	**BACE1**	
AT ^a^	0.020 ± 0.001	20.7 ± 2.2	10.0 ± 0.9	36.7 ± 3.3	1035
HAT ^a^	0.31 ± 0.02	>40	19.9 ± 0.2	24.7 ± 1.7	>129
MED	8.7 ± 0.3	>40	18.6 ± 1.3	35.9 ± 0.3	>4.59
Toloxatone	1.1 ± 0.03				
Clorgyline	0.007 ± 0.001				
Lazabemide		0.063 ± 0.015			
Pargyline		0.028 ± 0.004			
Donepezil			0.009 ± 0.002		
Tacrine			0.27 ± 0.02		
Quercetin				20.5 ± 0.6	

Results are expressed as mean and standard deviation from duplicate experiments. ^a^ Residual activity against hMAO-A and hMAO-B is 1-µM concentration. ^b^ SI values were calculated by hMAO-B/hMAO-A using IC_50_ values.

**Table 5:** Pharmacokinetic properties of the compounds predicted by in silico method.

Compound	GI Absorption	BBB Permeant	P-gp Substrate	CYP1A2 Inhibitor	CYP2C19 Inhibitor	CYP2C9 Inhibitor	CYP2D6 Inhibitor	CYP3A4 Inhibitor	Log *K_p_* (Skin Permeation)
**AT**	High	No	No	Yes	No	No	Yes	No	−6.18 cm/s
**HAT**	High	No	No	Yes	No	No	No	No	−5.82 cm/s
**MED**	High	Yes	No	No	No	No	No	No	−6.67 cm/s

GI: gastrointestinal; BBB: blood–brain barrier; P-gp: P-glycoprotein; CYP: cytochrome P450.

**Table 6:** Physicochemical parameters and Lipinski violations of the compounds.

Compound	Mw (g/mol)	cLog *P*	HBD	HBA	TPSA (Å²)	RB	Lipinski Violations
AT	274.23	1.83	4	6	111.13	0	0
HAT	258.23	2.17	3	5	90.9	0	0
MED	290.31	1.67	0	5	61.83	3	0

Mw: molecular weight; cLog P: consensus Log P ◦/w; HBD: H-bond donors; HBA: H-bond acceptors; TPSA: topological polar surface area; RB: rotatable bonds.

## Data Availability

Not applicable.

## Supplementary Materials

The followings are available online at https://www.mdpi.com/article/10.3390/jof7100876/s1. Figure S1. Percentage purity of AT (1); Figure S2. Percentage purity of HAT (2); Figure S3. Percentage purity of MED (3); Figure S4. 1H NMR spectrum of alternariol (AT) (1) in CD3OD; Figure S5. 13C NMR Spectrum of alternariol (AT) (1) in CD3OD; Figure S6. COSY Spectrum of alternariol (AT) (1) in CD3OD; Figure S7. HSQC Spectrum of alternariol (AT) (1) in CD3OD; Figure S8. HMBC Spectrum of alternariol (AT) (1) in CD3OD; Figure S9. 1H NMR spectrum of 5′-hydroxy-alternariol (HAT) (2) in DMSO-d6; Figure S10. 13C NMR Spectrum of 5′-hydroxy-alternariol (HAT) (2) in DMSO-d6; Figure S11. COSY Spectrum of 5′-hydroxy-alternariol (HAT) (2) in DMSO-d6; Figure S12. HSQC Spectrum of 5′-hydroxy-alternariol (HAT) (2) in DMSO-d6; Figure S13. HMBC Spectrum of 5′-hydroxy-alternariol (HAT) (2) in DMSO-d6; Figure S14. 1H NMR Spectrum of mycoepoxydiene (MED) (3) in DMSO-d6; Figure S15. 13C NMR Spectrum of mycoepoxydiene (MED) (3) in DMSO-d6; Figure S16. COSY Spectrum of mycoepoxydiene (MED) (3) in DMSO-d6; Figure S17. HSQC Spectrum of mycoepoxydiene (MED) (3) in DMSO-d6; Figure S18. HMBC Spectrum of mycoepoxydiene (MED) (3) in DMSO-d6; Figure S19. Plots of root mean square deviation (RMSD) during 1 ns MD simulation of the complex with (a) hMAO-A and AT, and (b) hMAO-B and AT.
