# Supplementary material for: Potent and Selective Inhibitors of Human Monoamine Oxidase A from an Endogenous Lichen Fungus Diaporthe mahothocarpus
Source: J Fungi (Basel). 2021 Oct 18;7(10):876. doi: 10.3390/jof7100876 (PMC8541017; doi:10.3390/jof7100876)
Supplement: Supplementary file 1 [file jof-07-00876-s001.zip › jof-1400135-supplementary.pdf]

Journal of
Fungi

MDPI

Supplementary Material

Potent and Selective Inhibitors of Human Monoamine Oxidase A from an Endogenous Lichen Fungus *Diaporthe mahothocarpus*

Geum Seok Jeong [1], Prima F. Hillman [2], Myung-Gyun Kang [3], Sungbo Hwang [3], Jong-Eun Park [1], Sang-Jip Nam [2], Daeui Park [3] and Hoon Kim [1,*]

[1] Department of Pharmacy, and Research Institute of Life Pharmaceutical Sciences, Sunchon National University, Suncheon 57922, Republic of Korea; fever41@naver.com (G.S.J.); nji8009@naver.com (J.I.N); park140201@naver.com (J.E.P); sungtae@scnu.ac.kr (S.T.Y)
[2] Department of Chemistry and Nanoscience, Ewha Womans University, Seoul 03760, Republic of Korea; primafitriah@gmail.com (P.F.H); sjnam@ewha.ac.kr (S.J.N)
[3] Department of Predictive Toxicology, Korea Institute of Toxicology, Daejeon 34114, Republic of Korea; myung-gyun.kang@kitox.re.kr (M.-G.K.); sungbo.hwang@kitox.re.kr(S.H.); daeui.park@kitox.re.kr (D.P.)
* Correspondence: Correspondence: hoon@sunchon.ac.kr; Tel.: +82-61-750-3751

Table of Contents

Figure S1. Percentage purity of AT (1)
Figure S2. Percentage purity of HAT (**2**)
Figure S3. Percentage purity of MED (**3**)
Figure S4. ^{1}H NMR spectrum of alternariol (AT) (**1**) in CD_3OD
Figure S5. ^{13}C NMR Spectrum of alternariol (AT) (**1**) in CD_3OD
Figure S6. COSY Spectrum of alternariol (AT) (**1**) in CD_3OD
Figure S7. HSQC Spectrum of alternariol (AT) (**1**) in CD_3OD
Figure S8. HMBC Spectrum of alternariol (AT) (**1**) in CD_3OD
Figure S9. ^{1}H NMR spectrum of 5'-hydroxy-alternariol (HAT) (**2**) in DMSO-d_6
Figure S10. ^{13}C NMR Spectrum of 5'-hydroxy-alternariol (HAT) (**2**) in DMSO-d_6
Figure S11. COSY Spectrum of 5'-hydroxy-alternariol (HAT) (**2**) in DMSO-d_6
Figure S12. HSQC Spectrum of 5'-hydroxy-alternariol (HAT) (**2**) in DMSO-d_6
Figure S13. HMBC Spectrum of 5'-hydroxy-alternariol (HAT) (**2**) in DMSO-d_6
Figure S14. ^{1}H NMR Spectrum of mycoepoxydiene (MED) (**3**) in DMSO-d_6
Figure S15. ^{13}C NMR Spectrum of mycoepoxydiene (MED) (**3**) in DMSO-d_6
Figure S16. COSY Spectrum of mycoepoxydiene (MED) (**3**) in DMSO-d_6
Figure S17. HSQC Spectrum of mycoepoxydiene (MED) (**3**) in DMSO-d_6
Figure S18. HMBC Spectrum of mycoepoxydiene (MED) (**3**) in DMSO-d_6
Figure S19. Plots of root mean square deviation (RMSD) during 1 ns MD simulation of the complex with (a) hMAO-A and AT, and (b) hMAO-B and AT

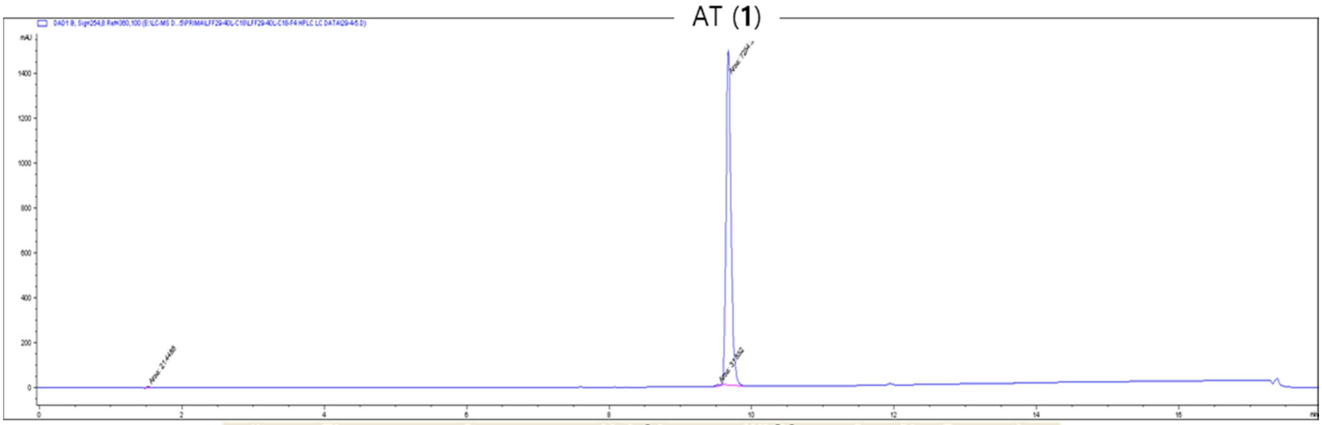

#	Time	Area	Height	Width	Area%	Symmetry
1	1.52	21.4	8	0.0448	0.294	0.827
2	9.526	31.9	9.7	0.0545	0.436	1.5
3	9.669	7254.4	1495.7	0.0808	99.271	0.806

Figure S1. Percentage purity of AT (**1**).

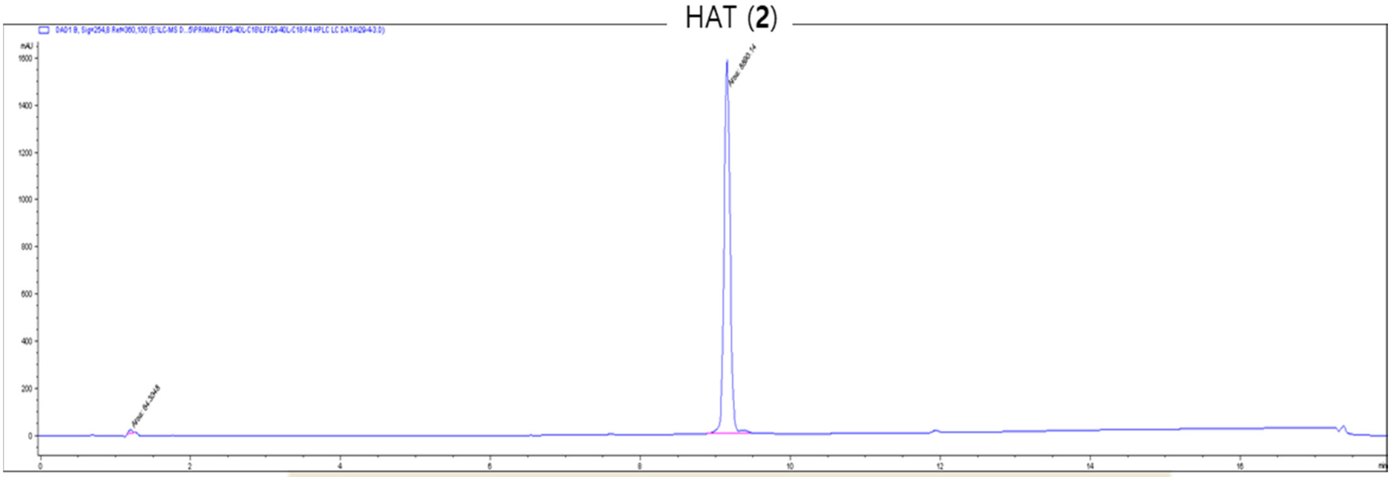

#	Time	Area	Height	Width	Area%	Symmetry
1	1.196	64.3	16.9	0.0633	0.718	1.018
2	9.153	8890.1	1585.9	0.0934	99.282	0.959

Figure S2. Percentage purity of HAT (**2**).

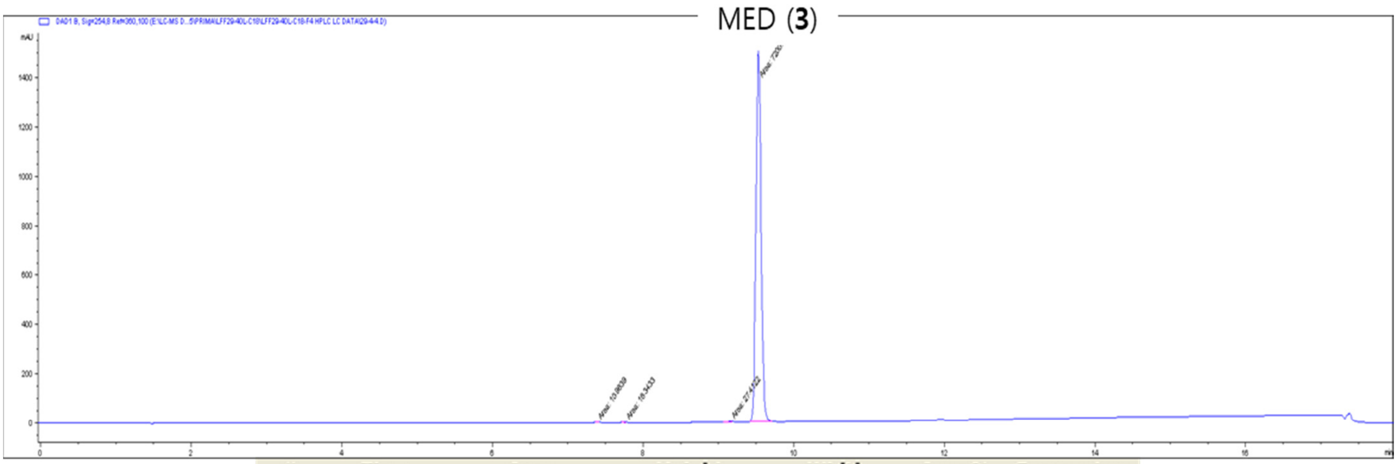

#	Time	Area	Height	Width	Area%	Symmetry
1	7.393	11	4.4	0.0414	0.151	1.291
2	7.762	16.3	6.7	0.0404	0.225	0.581
3	9.166	27.4	6.6	0.069	0.378	4.287
4	9.528	7200	1504.2	0.0798	99.246	0.906

Figure S3. Percentage purity of MED (**3**).

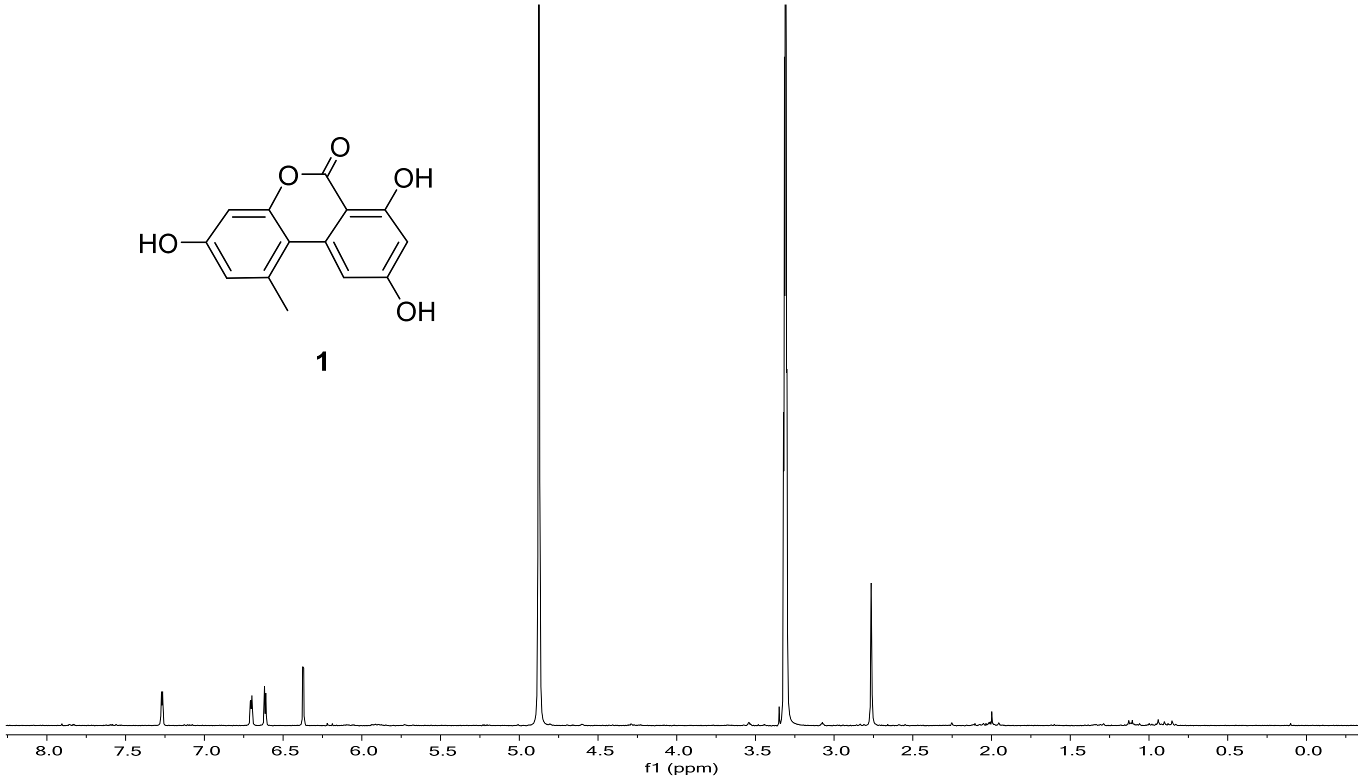

Figure S4. ^{1}H NMR spectrum of Alternariol (AT) (**1**) in CD_3OD.

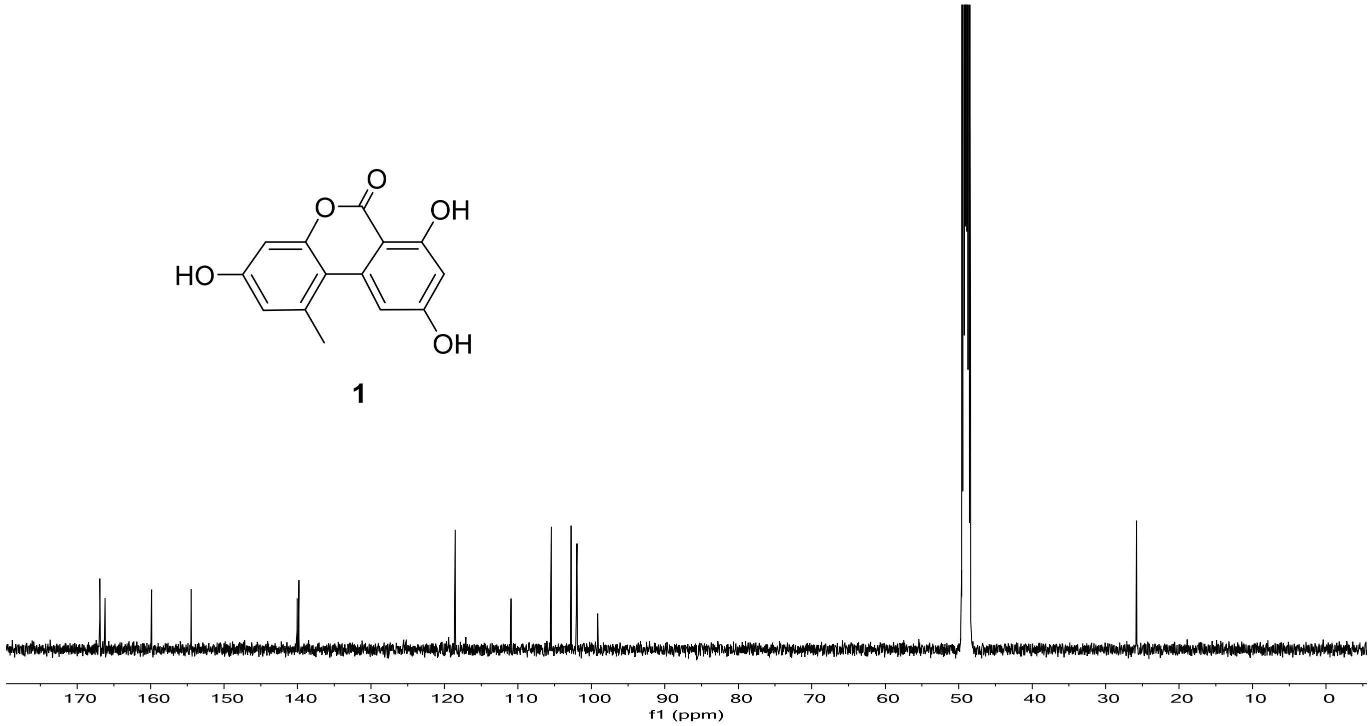

Figure S5. ^{13}C NMR spectrum of Alternariol (AT) (**1**) in CD_3OD.

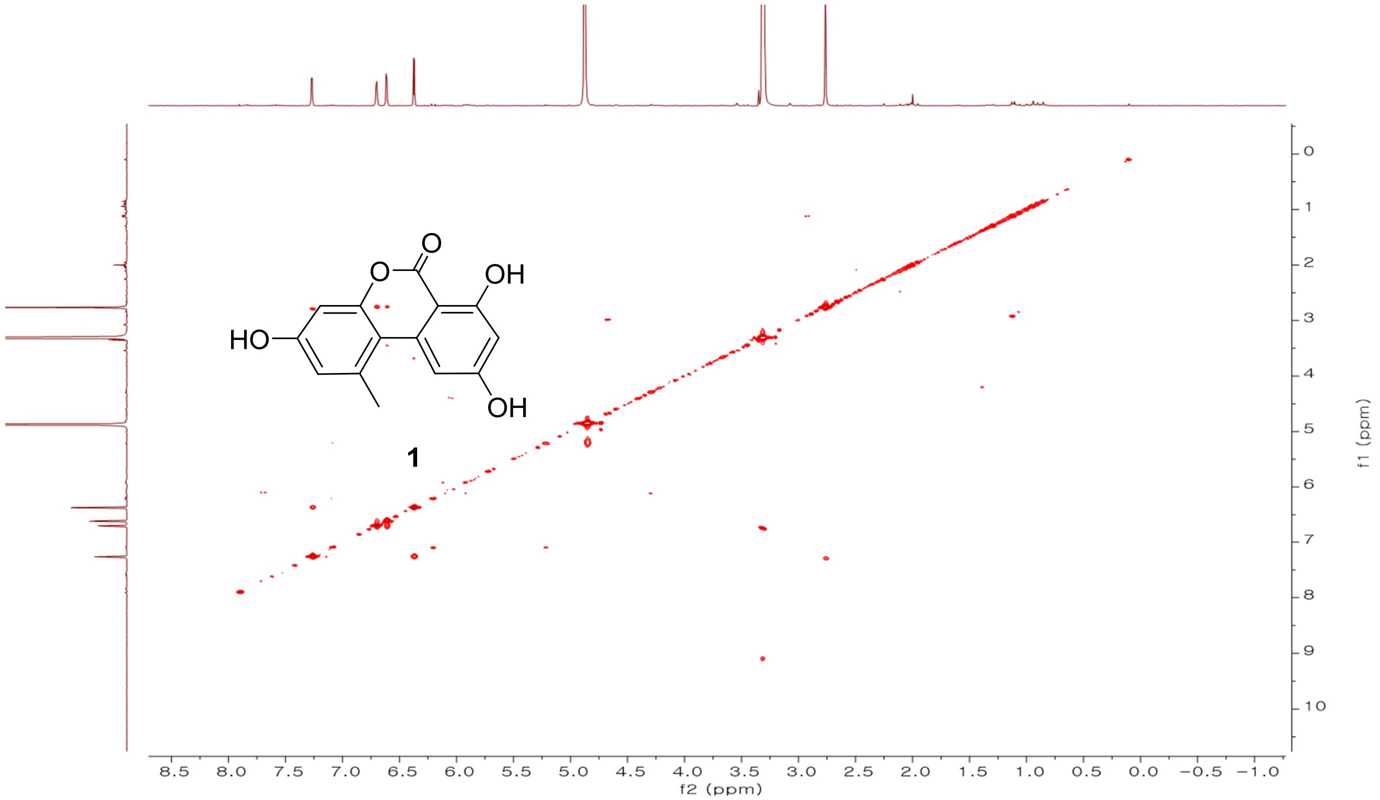

Figure S6. COSY spectrum of Alternariol (AT) (**1**) in CD_3OD.

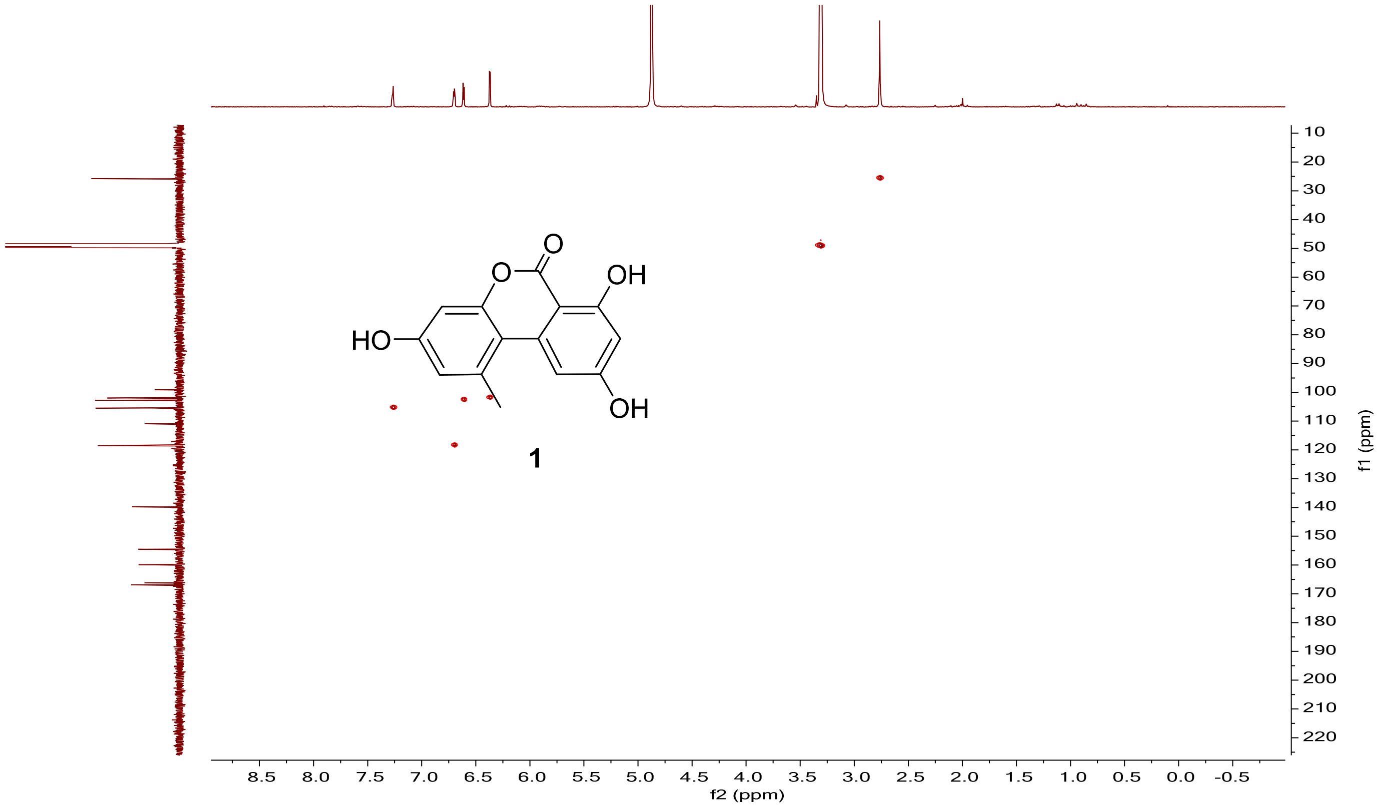

Figure S7. HSQC spectrum of Alternariol (AT) (**1**) in CD_3OD.

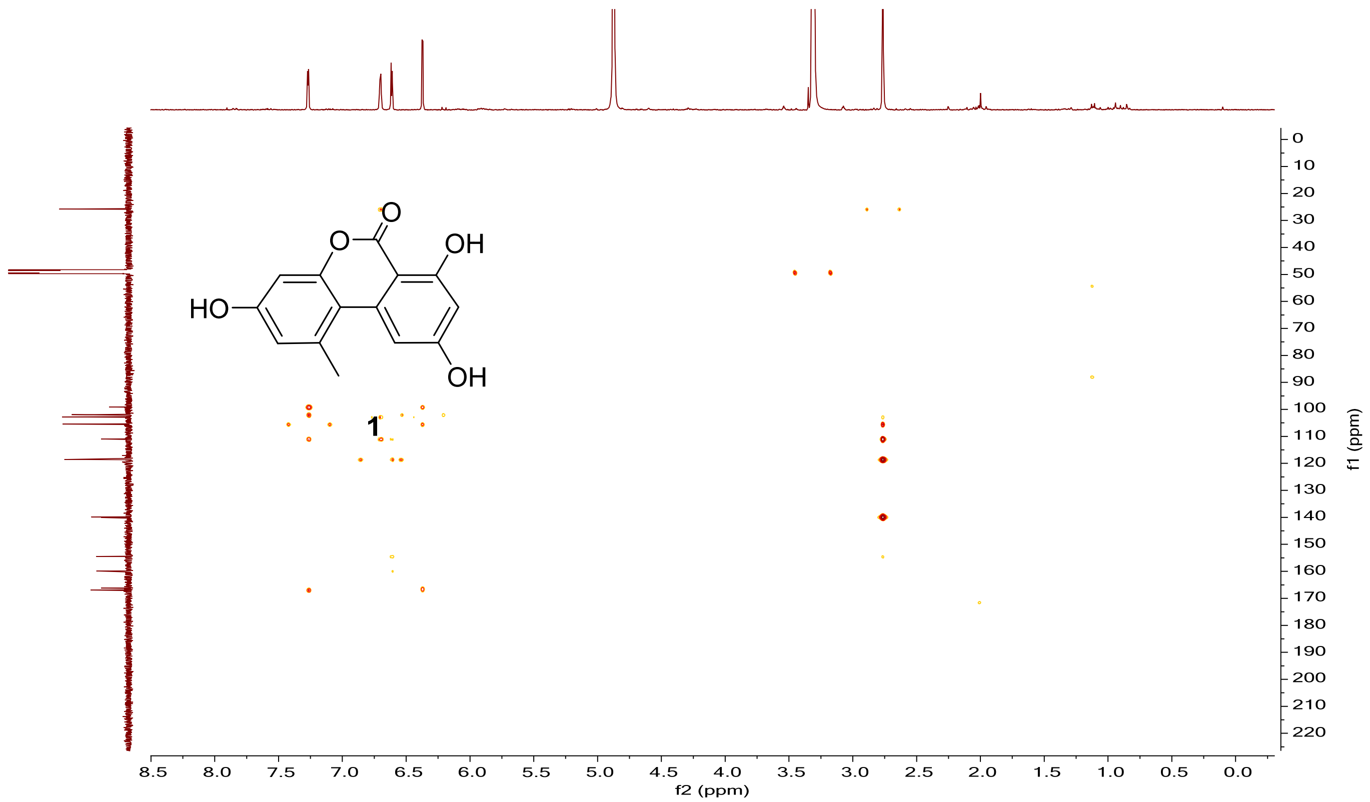

Figure S8. HMBC spectrum of Alternariol (AT) (**1**) in CD_3OD.

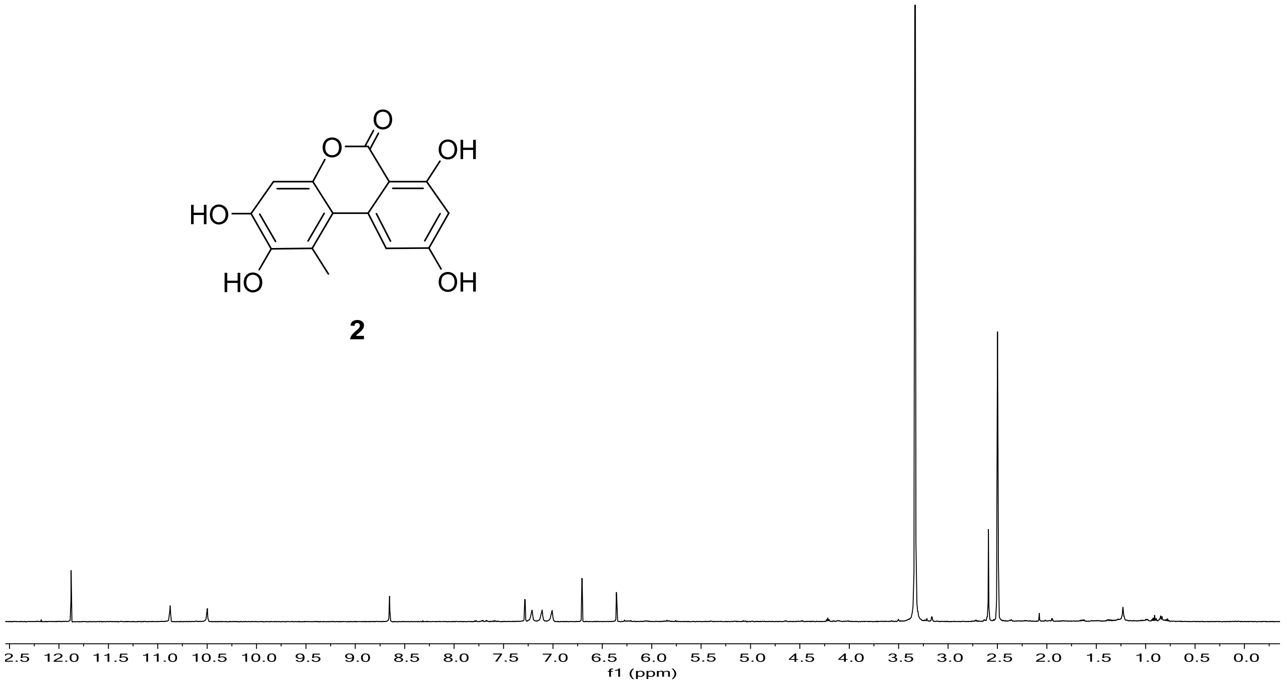

Figure S9. ^1H NMR Spectrum of 5'-hydroxy-alternariol (HAT) (**2**) in DMSO-d_6.

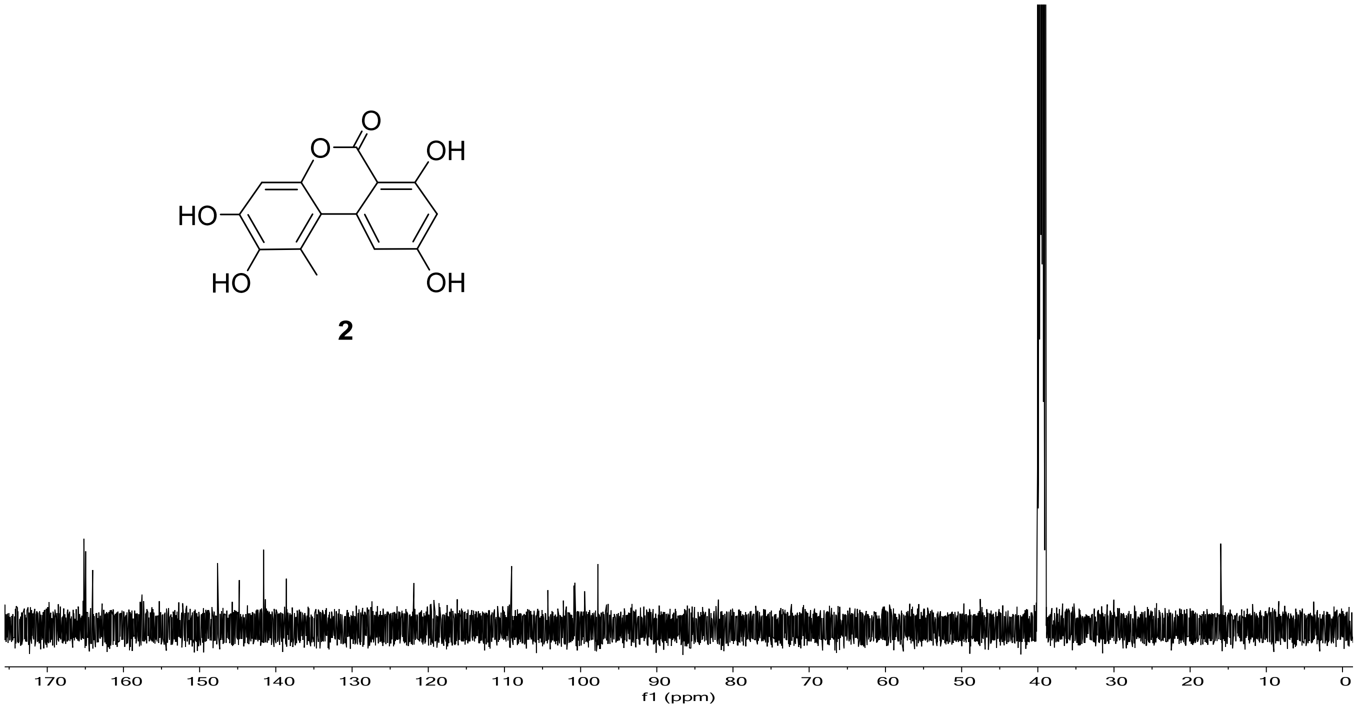

Figure S10. ^{13}C NMR Spectrum of 5'-hydroxy-alternariol (HAT) (**2**) in DMSO-*d*$_6$.

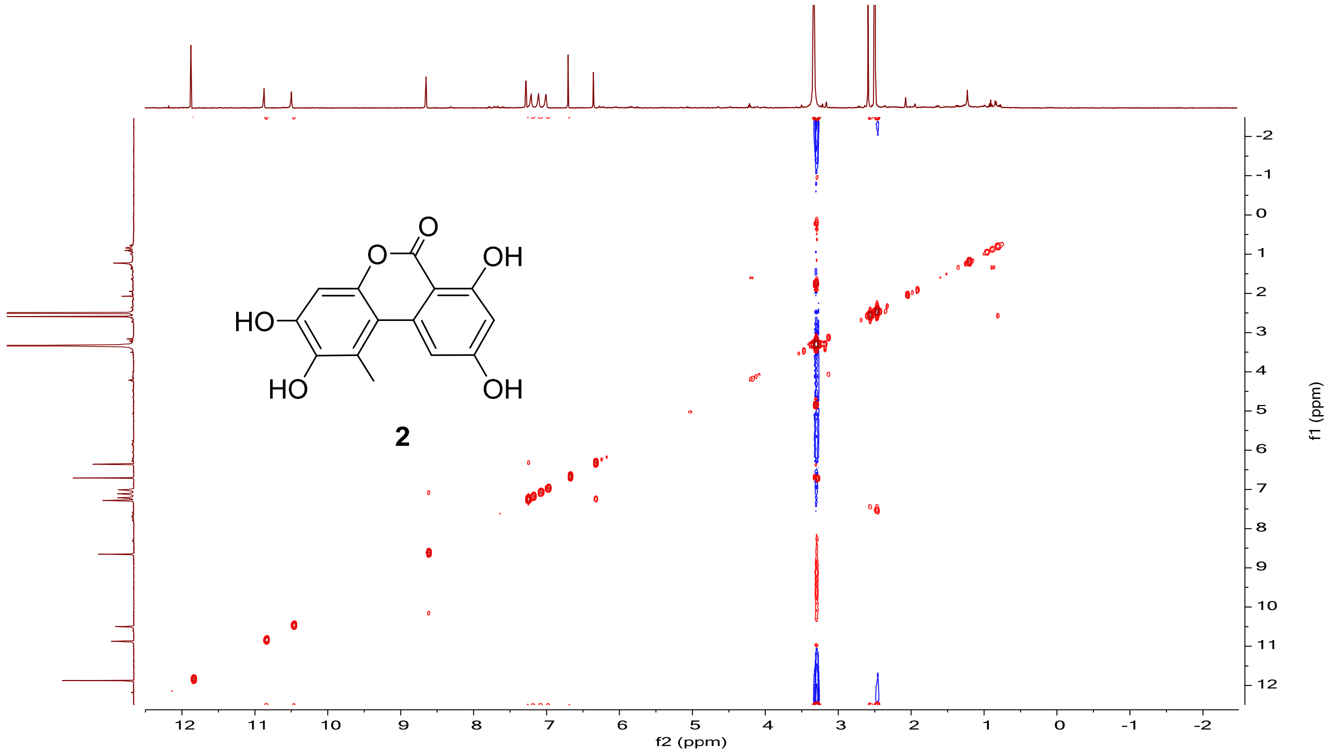

Figure S11. COSY Spectrum of 5'-hydroxy-alternariol (HAT) (**2**) in DMSO-*d*$_6$.

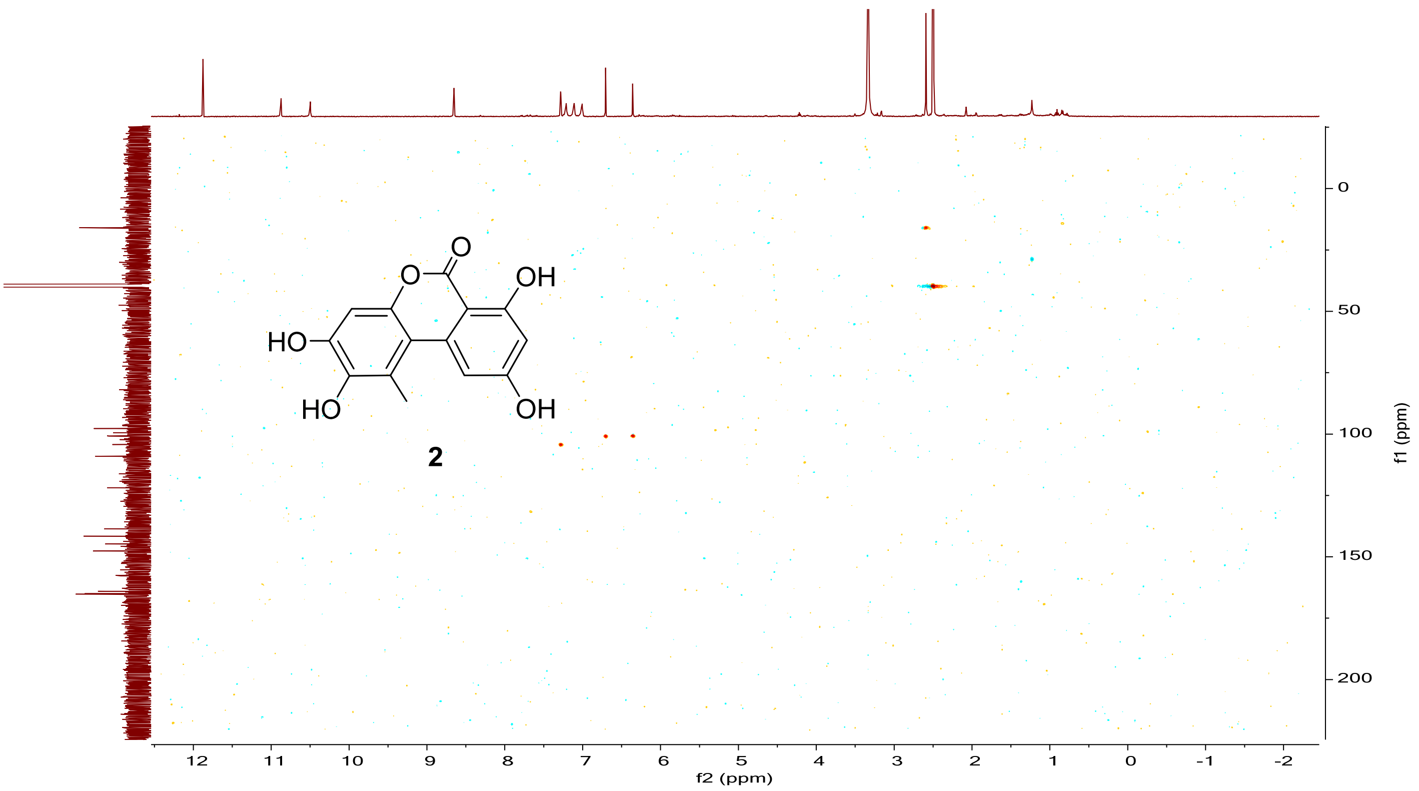

Figure S12. HSQC Spectrum of 5'-hydroxy-alternariol (HAT) (**2**) in DMSO-d_6.

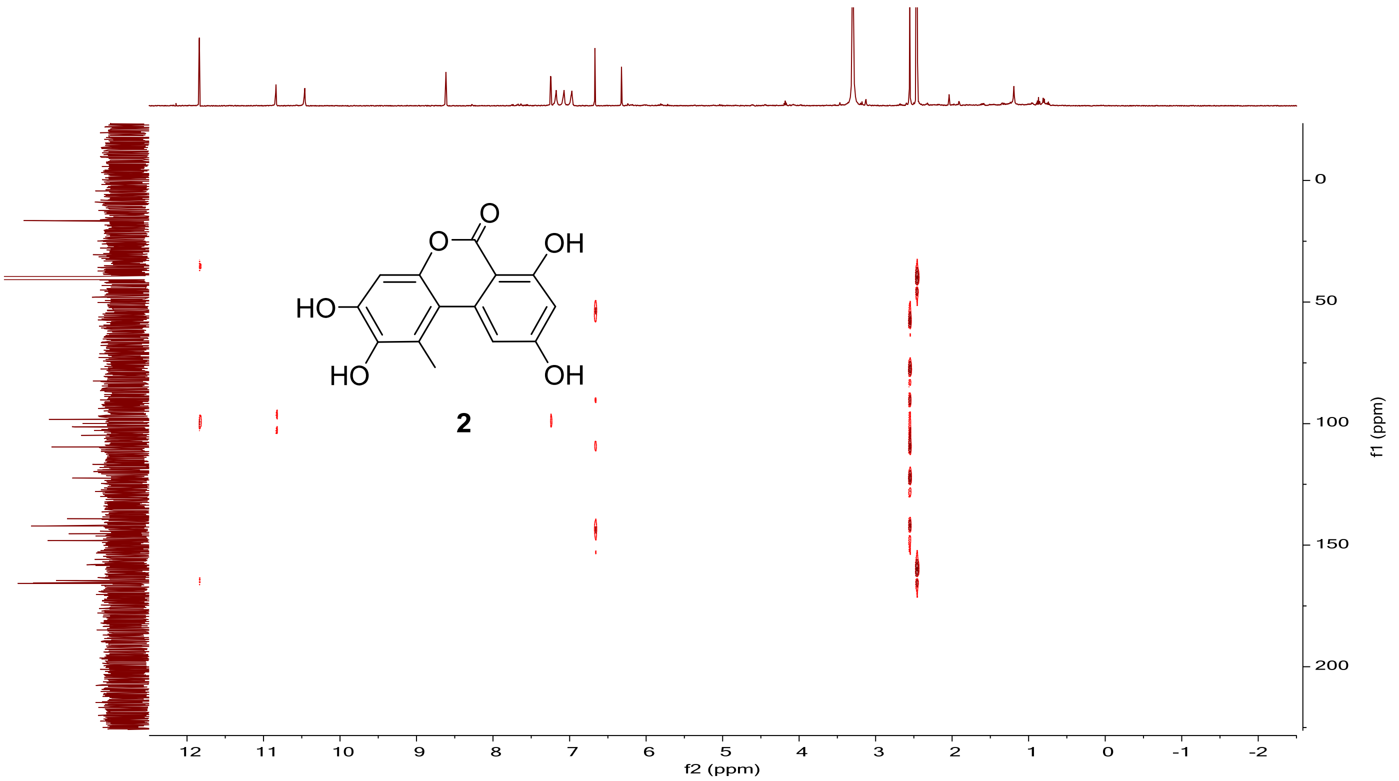

Figure S13. HMBC Spectrum of 5'-hydroxy-alternariol (HAT) (**2**) in DMSO-d_6.

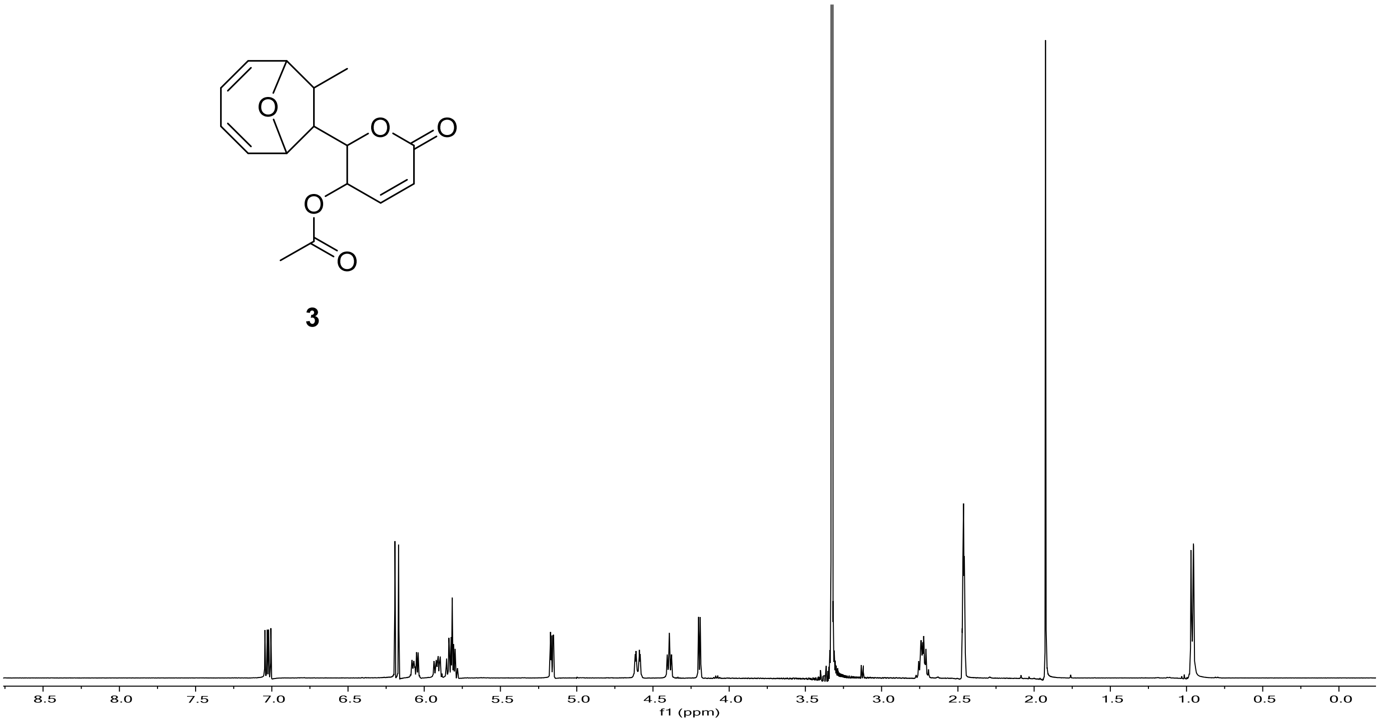

Figure S14. ^{1}H NMR spectrum of Mycoepoxydiene (MED) (**3**) in DMSO-d_6.

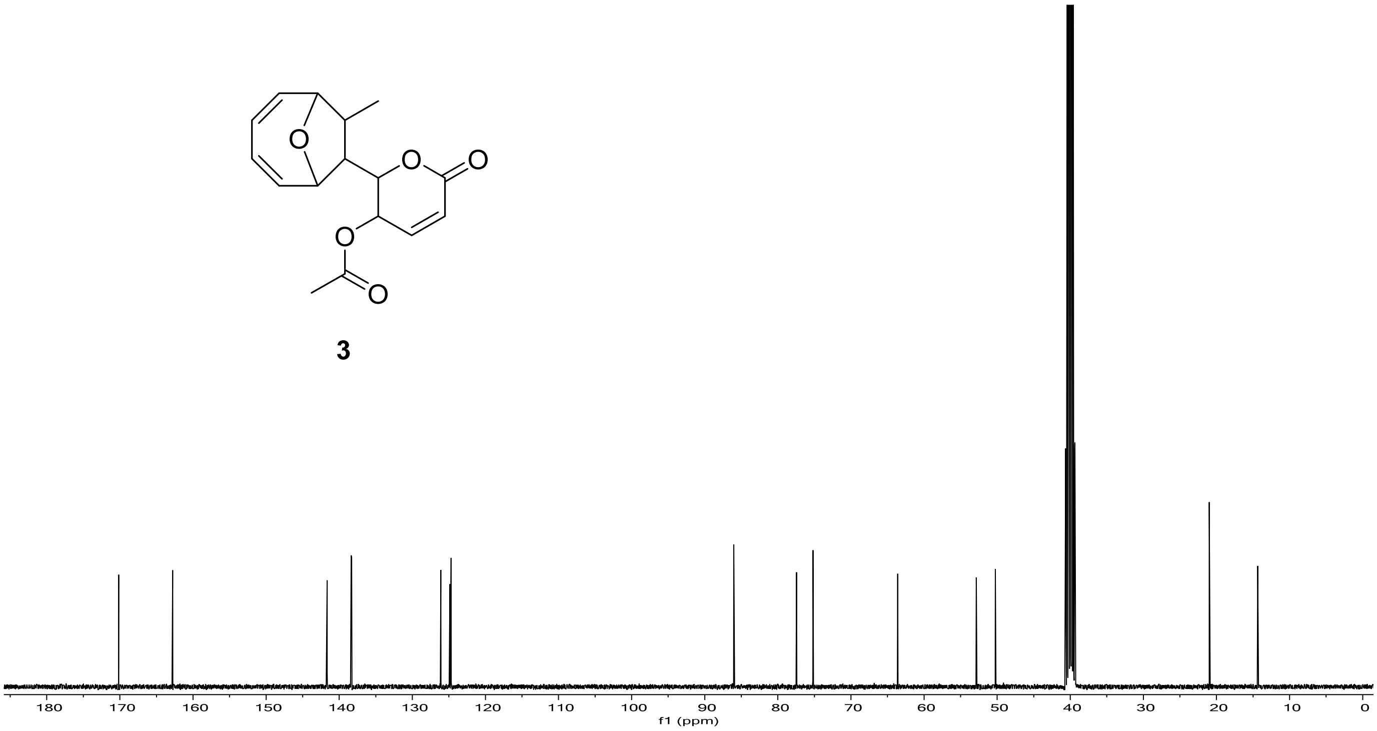

Figure S15. ^{13}C NMR Spectrum of Mycoepoxydiene (MED) (**3**) in DMSO-d_6.

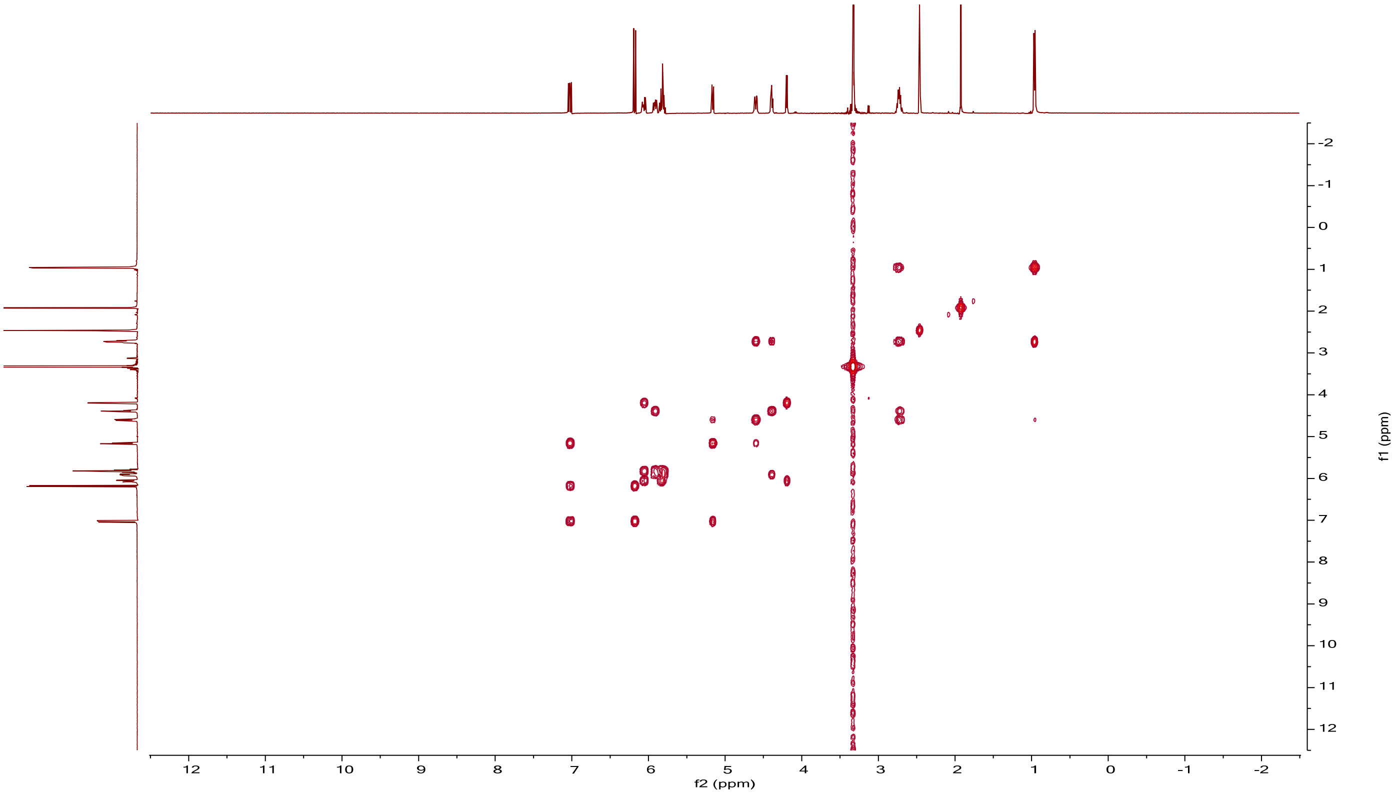

Figure S16. COSY Spectrum of Mycoepoxydiene (MED) (**3**) in DMSO-*d*6.

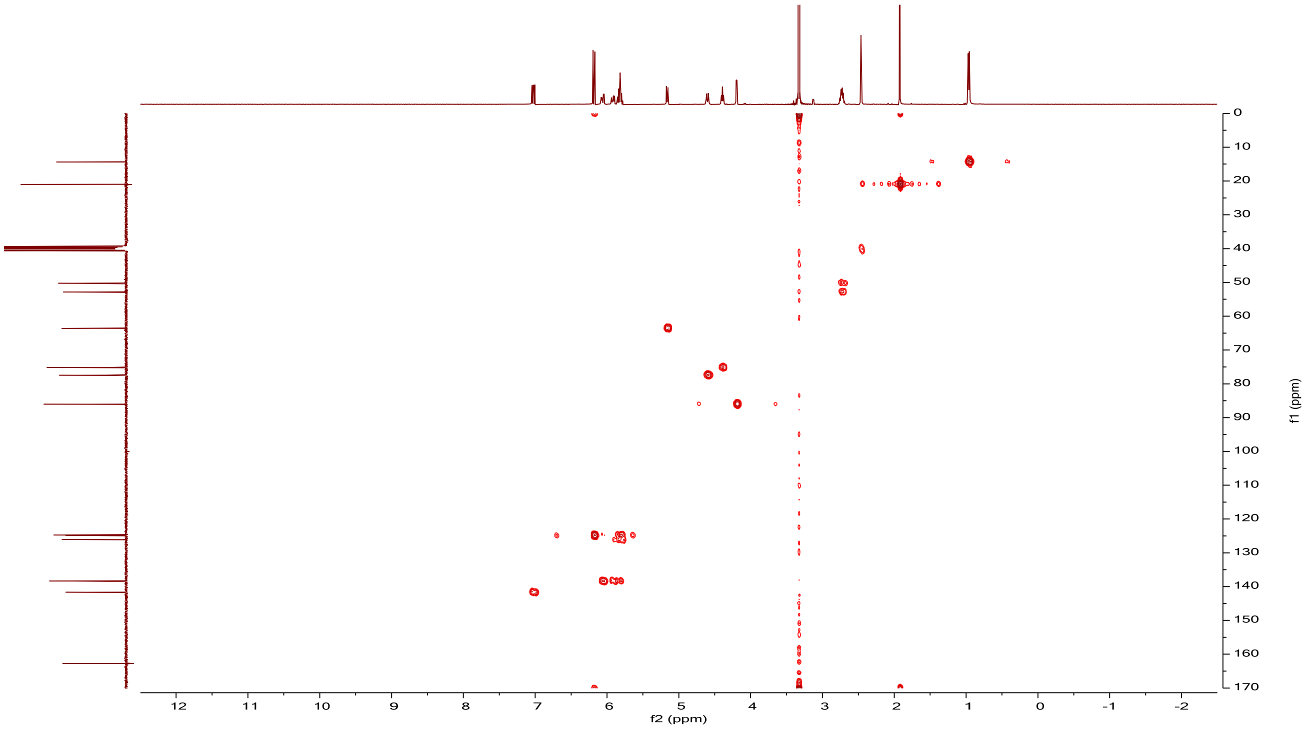

Figure S17. HSQC Spectrum of Mycoepoxydiene (MED) (**3**) in DMSO-*d*6.

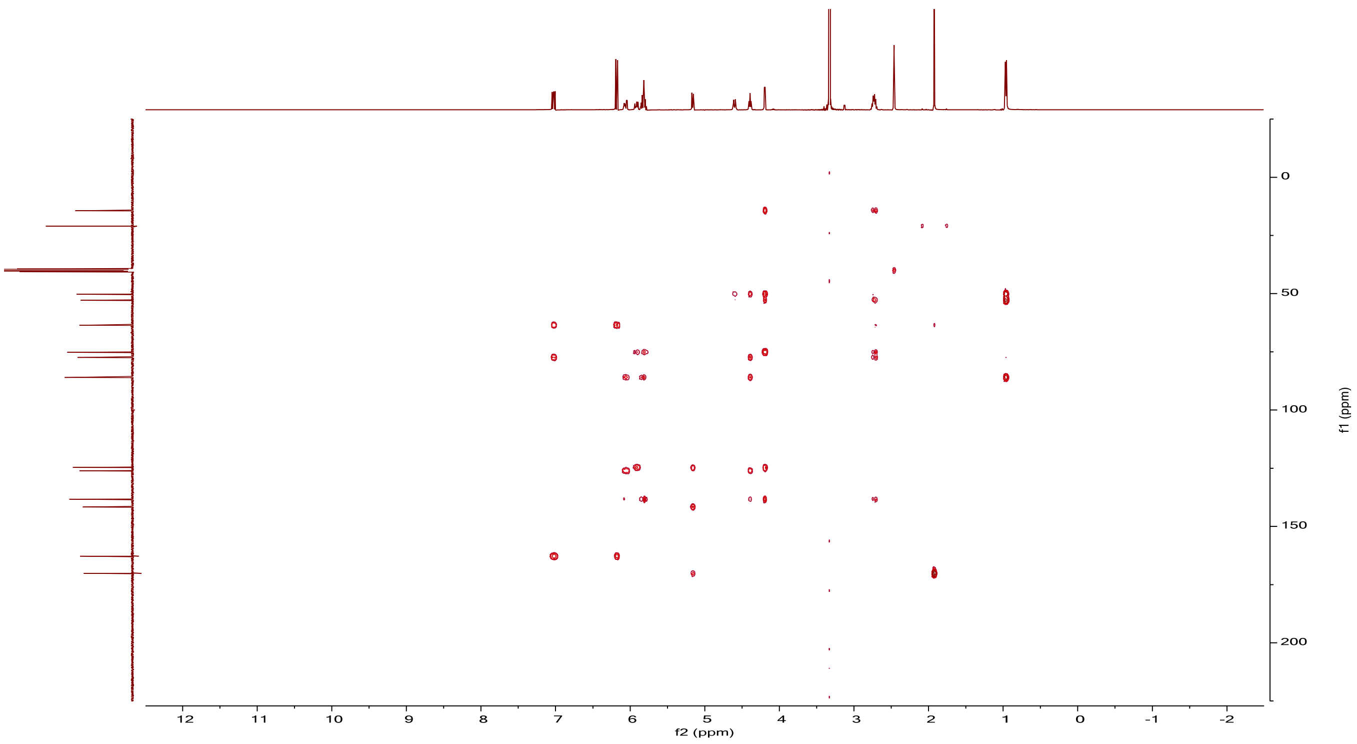

Figure S18. HMBC Spectrum of Mycoepoxydiene (MED) (**3**) in DMSO-d_6.

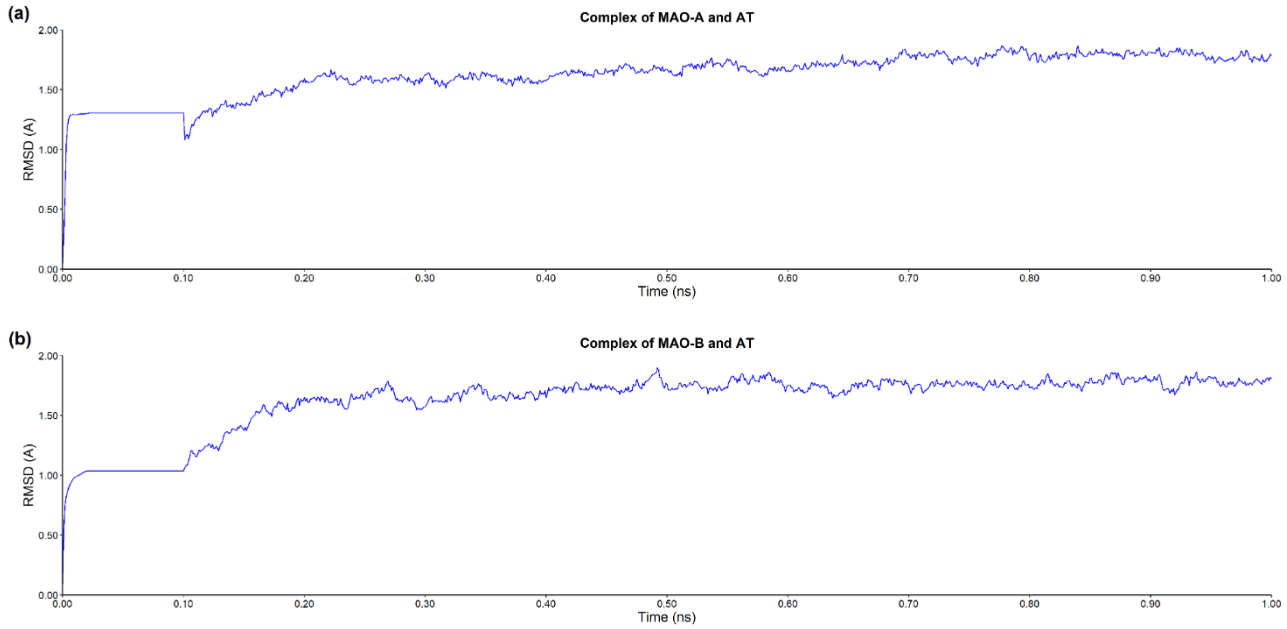

Figure S19. Plots of root mean square deviation (RMSD) during 1 ns MD simulation of (a) hMAO-A and (b) hMAO-B complexes with AT.
